# Acute tobacco smoke exposure exacerbates the inflammatory response to corneal wounds in mice via the sympathetic nervous system

**DOI:** 10.1038/s42003-018-0270-9

**Published:** 2019-01-24

**Authors:** Chengju Xiao, Mingjuan Wu, Jun Liu, Jianqin Gu, Xinwei Jiao, Dingli Lu, Jingxin He, Cuipei Lin, Yunxia Xue, Ting Fu, Hanqing Wang, Guang Wang, Xuesong Yang, Zhijie Li

**Affiliations:** 1 International Ocular Surface Research Center, Institute of Ophthalmology, Key Laboratory for Regenerative Medicine of the Ministry of Education, Jinan University, Guangzhou, China; 2 Henan Eye Institute, Henan Eye Hospital, Henan Provincial People’s Hospital, Henan University People’s Hospital, Zhengzhou, China; 3 Department of Ophthalmology, The First Affiliated Hospital, Jinan University, Guangzhou, China; 4 Department of Histology and Embryology, Jinan University Medical School, Guangzhou, China; 5 Section of Leukocyte Biology, Department of Pediatrics, Children’s Nutrition Research Center, Baylor College of Medicine, Houston, TX USA

## Abstract

Exposure to tobacco smoke is a major public health concern that can also affect ophthalmic health. Based on previous work demonstrating the important role of the sympathetic nervous system (SNS) in corneal wound repair, we postulated that acute tobacco smoke exposure (ATSE) may act through the SNS in the impairment of corneal wound repair. Here we find that ATSE rapidly increases the markers of inflammatory response in normal corneal limbi. After an abrasion injury, ATSE exaggerates inflammation, impairs wound repair, and enhances the expression of nuclear factor-κB (NF-κB) and inflammatory molecules such as interleukin-6 (IL-6) and IL-17. We find that chemical SNS sympathectomy, local adrenergic receptor antagonism, *NF-κB1* inactivation, and IL-6/IL-17A neutralization can all independently attenuate ATSE-induced excessive inflammatory responses and alleviate their impairment of the healing process. These findings highlight that the SNS may represent a major molecular sensor and mediator of ATSE-induced inflammation.

## Abstract (web summary)

Chengju Xiao et al. find that acute tobacco smoke exaggerates inflammation in recently wounded mouse corneas, thereby impairing wound repair. They show that the effect of tobacco smoke is mediated by activation of the sympathetic nervous system.

## Introduction

The cornea accounts for one-third of the entire visual system’s refractive power. Corneal wound healing is one of the most common patient needs in a vision clinic^1,2^. Rapid and complete repair is the ideal scenario for infection prevention and rapid vision recovery. It has been suggested that mechanical corneal epithelium abrasion induces a highly programmed reaction involving reepithelialization, proliferation, inflammation, and late-stage extracellular matrix remodeling^2–8^. The repair process is influenced by both intrinsic and extrinsic factors, but the impact of the external environment is crucial.

Tobacco smoke exposure, an environmental harm encountered in everyday life, is considered a major public health problem. Recently, the dangers of acute tobacco smoke exposure (ATSE), such as respiratory toxicity, have become of increasing concern^9–11^. In addition to its toxicity to the respiratory, hematopoietic, and cardiovascular systems, visual system damage by ATSE has also received close attention. ATSE majorly impacts ocular surface health and is considered the primary causative agent of ocular surface abnormalities such as dry eye^12^, conjunctival inflammation, and conjunctiva squamous metaplasia due to the local accumulation of reactive oxygen species (ROS) or increased inflammation^13–19^. Studies have shown that ATSE can profoundly affect corneal wound repair and exaggerate the inflammatory response^20–22^. However, the molecular mechanism for this impaired wound healing is not well understood.

Tobacco smoke is a complex, dynamic chemical mixture containing more than 4000 different substances, so it can harm the human body’s physiological mechanisms in multiple ways^23–25^. The corneas are heavily innervated by small-diameter sensory C-fibers sensitive to environmental stresses such as ATSE^26^. Functional loss and sensitization of corneal sensory nerve fibers significantly alter corneal wound healing and inflammatory response^27,28^. However, we recently found that the mouse cornea, especially the corneal limbus and peripheral cornea, is also innervated by sympathetic nervous system (SNS) fibers^29^. More importantly, interaction with immune cells gives the SNS a unique role in corneal wound closure^29^.

The SNS is distributed throughout all the body’s organs and tissues, including the cornea^30,31^, and SNS afferent nerve fibers are sensitive to chemical stimuli. After tobacco smoke exposure, human SNS activity is rapidly amplified and manifests as increased plasma catecholamines (CChs; e.g., norepinephrine, epinephrine, and dopamine) levels, blood pressure, and heart rate^32–34^. Recent research has shown that the SNS regulates many immune system functions, primarily through α-adrenergic receptor and/or β-adrenergic receptor (β-AR) signaling pathways in immune cells^35–38^. These pathways might directly or indirectly alter inflammatory cell recruitment^39,40^ and the production of inflammatory cytokines, for example, interleukin-6 (IL-6) and IL-17A. The SNS may activate the nuclear factor-κB (NF-κB) pathway, through mechanisms not yet fully understood, to positively or negatively regulate the immune system and inflammatory responses depending on different environments and tissues^41,42^. However, whether and how increased SNS tone after ATSE affects corneal wound healing and inflammation has yet to be established.

Given previous findings, we hypothesized that ATSE impairs corneal wound healing and exacerbates inflammation by triggering SNS overactivation. The consequent increase in CChs stimulates ARs on immune cells infiltrating the wound site and induces NF-κB and inflammatory molecule (e.g., IL-6 and IL-17) expression, impeding healing. We predicted that chemically sympathectomizing the SNS, inhibiting ARs with antagonists, neutralizing IL-17A and IL-6 with antibodies, or genetically inactivating NF-κB would each be able to alleviate ATSE-induced corneal healing impairment post injury. Our findings highlight SNS involvement in the pathogenesis of ATSE-induced corneal healing impairment in mice, elucidate the mechanistic role of adrenergic signaling, NF-κB, IL-6, and IL-17A in this phenomenon, and reveal potential therapeutic opportunities for ameliorating ATSE-associated effects.

## Results

### ATSE delays wound repair and exacerbates inflammation

**Fig. 1:**
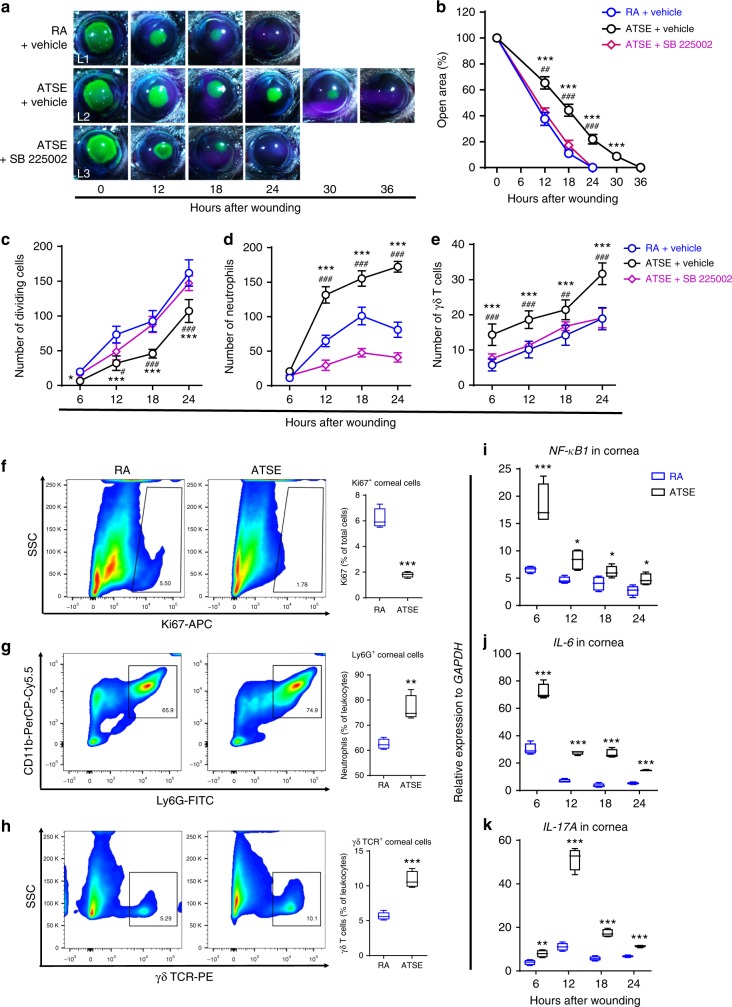
Acute tobacco smoke exposure (ATSE) impairs corneal wound healing and exacerbates inflammation. **a** Representative images of open corneal wounds (revealed by a topical fluorescein solution) and visible wound closure over time. **b** Percent decrease in open wound area over time post-wounding (two-way RM ANOVA, interaction *p* < 0.0001, Bonferroni’s multiple comparisons test, *n* = 6 corneas per time point in each group). **c** Change over time in the number of dividing epithelial cells after wounding (two-way AVOVA, interaction *p* = 0.00027, Sidak’s multiple comparisons test, *n* = 6 corneas per time point in each group). **d** Neutrophil influx into cornea over time after corneal abrasion (two-way AVOVA, interaction *p* < 0.0001, Sidak’s multiple comparisons test, *n* = 6 corneas per time point in each group). **e** γδ T cell influx into wounded cornea over time after corneal abrasion (two-way AVOVA, interaction *p* = 0.0118, Sidak’s multiple test, *n* = 6 corneas per time point in each group). **f**–**h**) Representative flow cytometry plots of CD11b^+^ Ly6G^+^ neutrophils (Student’s *t* test, *p* < 0.0001, *n* = 4), GL3^+^ γδ T cells (Student’s *t* test, *p* = 0.0022, *n* = 4), and Ki-67^+^ cells (Student’s *t* test, *p* = 0.0003, *n* = 4), quantified by flow cytometric analysis of cells from wounded corneas of mice treated with either RA or ATSE at 18 h post wounding (20 pooled corneas for each batch, four independent groups). *SSC* side scatter. **i**–**k** Relative expression of *NF-kB*, *IL-6*, and *IL-17A* measured by qRT-PCR in whole-corneal mRNA at 6, 12, 18, and 24 h after abrasion (Student’s *t* test, *p < 0.05, **p < 0.01, ***p < 0.001, *n* = 4). *Statistically significant difference between RA and ATSE groups. ^#^statistically significant difference between ATSE and ATSE + SB 225002 groups. *^, #^*p* < 0.05; **^, ##^*p* < 0.01; ***^, ###^*p* < 0.001

To characterize ATSE effects on corneal wound repair, we designed a modified ATSE protocol^43^. Then, in C57BL/6J mice breathing room air (RA) and mice subjected to ATSE at 12-h intervals (Supplementary Figure 1), we compared corneal epithelial closure and the division and influx of neutrophils and γδ T cells into the wounded cornea after epithelial abrasion. In the RA group, reepithelialization was usually complete 24 h post abrasion (Fig. 1a (L1), b); in the ATSE group, reepithelialization finished only 36 h post abrasion (Fig. 1a (L2), b). To investigate the effect of ATSE on epithelial cell division (Supplementary Figure 2A) after abrasion, we used our previously described method for counting epithelial cell divisions to analyze kinetics of dividing cells^8,44^. The number of dividing epithelial cells in ATSE-treated mice was significantly lower at 6, 12, 18, and 24 h after injury compared to that in RA control mice (Fig. 1c). To understand the impact of ATSE on the post-wounding corneal inflammatory response, we used previously described methods for counting neutrophils (Supplementary Figure 2B) and measuring the γδ T cells (Supplementary Figure 2C) influx into the wounded cornea^3,45^. Compared to the RA group, the ATSE group showed a markedly enhanced neutrophil influx (Fig. 1d) and significantly higher number of γδ T cells (Fig. 1e) trafficking to the wounded cornea at 6, 12, 18, and 24 h after injury. Flow cytometric analyses of corneal cells harvested 18 h after wounding confirmed these observations and showed fewer Ki-67^+^ proliferative cells present (Fig. 1f–h, respectively). Finally, we compared the gene expression of *NF-κB1* and the proinflammatory molecules *IL-6* and *IL-17A*, highly relevant to smoke exposure-induced inflammation^46–48^, in the whole-corneal messenger RNA (mRNA) of RA- and ATSE-treated animals. All three genes were transcribed at significantly higher rates in ATSE-treated animals (Fig. 1j–l, respectively). Taken together, and consistent with previous reports^20–22^, our data suggest that ATSE impairs wound healing and exacerbates inflammatory response after murine corneal injury.

The level of the chemokine (C-X-C motif) ligand 1/KC (CXCL1/KC) rapidly increases locally post abrasion^44^. Its receptor CXCR2 is responsible for neutrophil chemotaxis in the inflamed or wounded tissue^49^. To determine whether excessive inflammation was important in ATSE-impaired corneal healing, mice received intraperitoneal (i.p.) injections of the CXCR2 antagonist SB 225002 (1 mg kg^−1^) or a vehicle 5 min before corneal abrasion. We then observed the effect of neutrophil decrease on wound repair. As expected, the number of neutrophils and γδ T cells significantly decreased (Fig. 1d, e). These mice showed reepithelialization and epithelial cell division comparable to RA animals (Fig. 1a (L3), b, c). Our results suggest that the suppression of excessive neutrophil migration into wounded corneas can improve the ATSE-impaired repair process.

### ATSE enhances mobilization of immune cells via the SNS

**Fig. 2:**
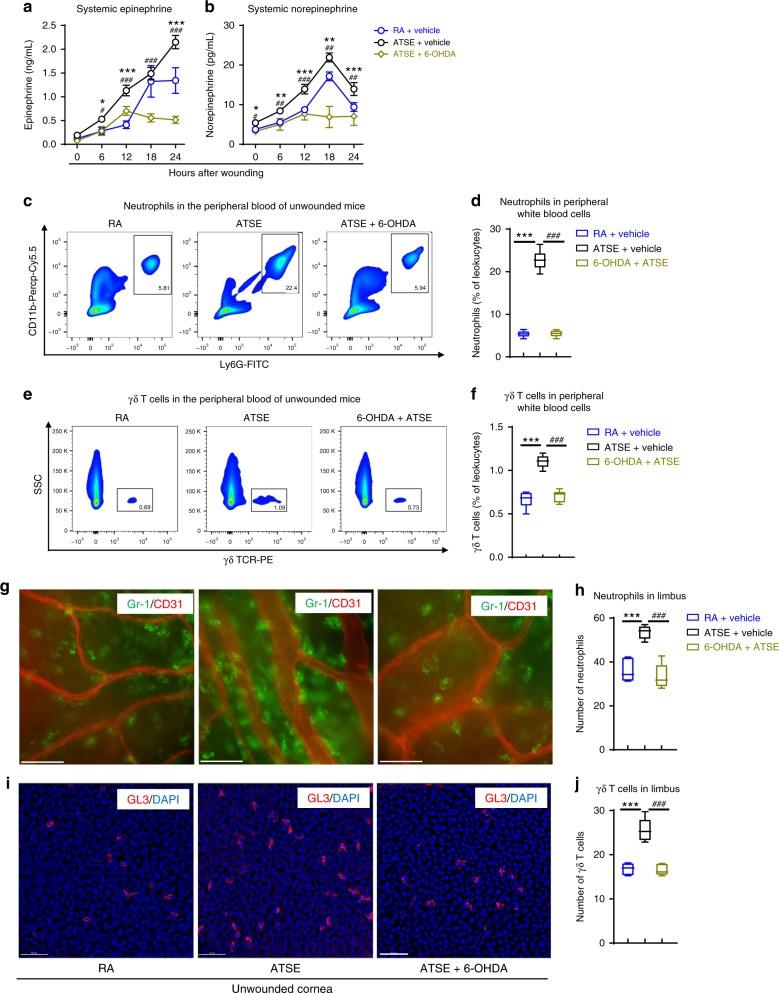
ATSE enhances SNS signaling activity and mobilization of neutrophils and γδ T cells into the circulation and corneal limbus. **a** ELISA-measured plasma epinephrine concentration after corneal abrasion in RA, ATSE, and ATSE + 6-OHDA treatments (two-way AVOVA, interaction *p* < 0.0001, Sidak’s multiple comparisons test, *n* = 4). **b** ELISA-measured plasma norepinephrine concentration after corneal abrasion in RA, ATSE, and ATSE + 6-OHDA treatments (two-way AVOVA, interaction *p* < 0.0001, Sidak’s multiple comparisons test, *n* = 4). **c** Representative flow cytometric quantification of CD11b^+^Ly6G^+^ neutrophils in peripheral blood of mice without corneal abrasion 1 h after RA, ATSE-only, and 6-OHDA + ATSE treatments. **d** Cumulative neutrophil (CD11b^+^Ly6G^+^) percentage among total leukocytes in peripheral blood 1 h after ATSE (one-way ANOVA, Tukey's post hoc, *p* < 0.0001 for RA vs. ATSE and ATSE vs. ATSE + 6-OHDA, *n* = 6 mice per group). **e** Representative flow cytometric quantification of γδ T cells in peripheral blood of mice without corneal abrasion 1 h after RA, ATSE-only, and 6-OHDA + ATSE. **f** Cumulative percentage of γδ T cells (GL3^+^) among total leukocytes in peripheral blood 1 h after ATSE (one-way ANOVA, Tukey's post hoc, *p* < 0.0001 for RA vs. ATSE and ATSE vs. ATSE + 6-OHDA, *n* = 6 mice per group). **g** Representative Ly6G^+^ neutrophil (labeled with FITC-Gr1: green) recruitment around limbal vessels (epithelial cell marker CD31: red) of mouse without corneal abrasion 1 h after RA, ATSE-only, and 6-OHDA + ATSE treatments; ×40, scale bars 40μm. **h** Quantification of neutrophil recruitment to corneal limbus 1 h after RA, ATSE-only, and 6-OHDA + ATSE treatments (one-way ANOVA, Tukey's post hoc test, *p* < 0.0001 for RA vs. ATSE and ATSE vs. ATSE + 6-OHDA, *n* = 5). **i** Representative GL3^+^ γδ T cell (anti-GL3: red) accumulation in limbus 1 h after RA, ATSE-only, and 6-OHDA + ATSE treatments; ×40, scale bars 40μm. **j** Quantification of γδ T cell recruitment to corneal limbus 1 h after RA, ATSE-only, and 6-OHDA + ATSE treatments (one-way ANOVA, Tukey's post hoc, *p* < 0.0001 for RA vs. ATSE, ATSE vs. ATSE + 6-OHDA, *n* = 5); *^, #^*p* < 0.05; ^**, ##^*p* < 0.01; ***^, ###^*p* < 0.001, comparing groups as indicated

The above results showed that ATSE delays corneal wound repair through excessive inflammation; the SNS is an important regulator of immune and inflammatory responses^41,50^. In human studies, exposure to tobacco smoke quickly increased SNS activity^33,34,51^. Based on this evidence, we hypothesized that exposure to tobacco smoke may influence immune status through SNS overactivation. To characterize the kinetics of sympathetic neural signaling in a mouse model after ATSE and corneal wounding, we measured epinephrine and norepinephrine levels in the plasma after corneal injury in mice subjected to ATSE for only two 90-min sessions (before and 12 h after abrasion). ATSE animals showed a significant systemic increase in epinephrine and norepinephrine compared to RA animals (Fig. 2a). To confirm whether these increased levels derived from the overactivated SNS after ATSE, we chemically ablated peripheral nervous system innervation in these animals by pretreatment with 6-hydroxydopamine (6-OHDA), a chemical selectively toxic to the sympathetic system^52^. The ablated mice showed levels of epinephrine and norepinephrine in the plasma similar to those of normal mice in the RA group (Fig. 2a, b). Together, these results suggest that ATSE strongly contributes to SNS overactivation.

Artificial SNS activation by stress and epinephrine infusion rapidly induces leukocytosis, a blood white cell count above the normal range^53,54^. To confirm the effect of ATSE-induced SNS overactivation on the immune status of normal, unwounded mice, flow cytometry was used to detect phase changes in peripheral blood. The number of CD45^+^ cells in mouse peripheral blood significantly increased 1 h after ATSE treatment (Supplementary Figure 3). This finding was in line with the leukocytosis seen in humans following smoke exposure^55,56^. To identify the cell subtypes in the CD45^+^ leukocytes, we used different cell surface markers to specifically label neutrophils and γδ T cells. The number of CD11b^+^ Ly6G^+^ neutrophils (Fig. 2c, d) and GL3^+^ γδ T cells (Fig. 2e, f) were significantly higher in ATSE mice. However, the number of CD11b^+^ Ly6G^+^ neutrophils and GL3^+^ γδ T cells were stable in 6-OHDA-pretreated animals (Fig. 2c, d and 2e, f, respectively). Finally, to determine ATSE effects on the distribution of immune cells, we used corneal whole mounts to observe the presence and number of Ly6G^+^ neutrophils and GL3^+^ γδ T cells in normal corneal tissue. We found that neutrophils (Fig. 2g, h) and γδ T cells (Fig. 2i, j) rapidly recruited to the limbal vascular network and epithelium in unwounded corneas 1 h post ATSE. Conversely, in 6-OHDA-pretreated animals, ATSE stimulation did not significantly increase neutrophil or γδ T cell recruitment to the corneal limbus (Fig. 2g–j). Altogether, these findings provide compelling evidence that ATSE can rapidly alter the mouse immunological environment at systemic and local levels through SNS activation.

### Local epinephrine induces inflammatory exacerbation

**Fig. 3:**
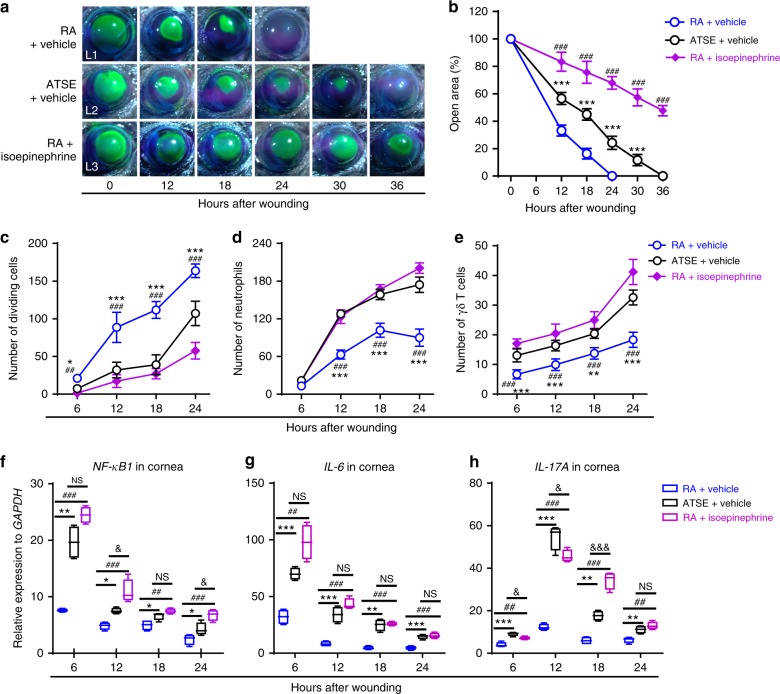
Topical administration of a β-adrenergic agonist in the RA group worsens corneal wound healing and inflammation. **a** Representative images of open corneal wounds (revealed by a topical fluorescein solution) and visible wound closure over time. **b** Percent decrease in open wound area over time post wounding (two-way RM ANOVA, interaction *p* < 0.0001, Bonferroni’s multiple comparisons test, *n* = 6 corneas per time point in each group). **c** Number of dividing epithelial cells over time after wounding (two-way AVOVA, interaction *p* < 0.0001, Sidak’s multiple comparisons test, *n* = 6 corneas per time point in each group). **d** Neutrophil influx into the wounded area (Field 4 and 4ʹ in Supplementary Figure 4) over time after corneal abrasion (two-way ANOVA, interaction *p* < 0.0001, Sidak’s multiple comparisons test, *n* = 6 corneas per time point in each group). **e** γδ T cell influx into the wounded cornea over time after corneal abrasion (two-way ANOVA, interaction *p* < 0.0001, Sidak’s multiple comparisons test, *n* = 6 corneas per time point in each group). **f**–**h** Relative expression of *NF-kB*, *IL-6*, and *IL-17A* measured using mRNA isolated from whole wounded corneas of the RA, ATSE, and RA + isoepinephrine groups at 6, 12, 18, and 24 h after corneal abrasion (one-way ANOVA, Tukey's post hoc, RA vs. ATSE, *p < 0.05, **p < 0.01, ***p < 0.001; RA vs. RA + isoepinephrine, ^##^*p* < 0.01, ^###^*p* < 0.001, ATSE vs. RA + isoepinephrine, & *p*< 0.05, &&& *p*<0.001, *n* = 4). Symbols denoting statistical significance: * compares RA and ATSE groups; ^#^ compares RA and RA + isoepinephrine groups; ^&^ compares ATSE and RA + isoepinephrine groups. The number of symbols used denotes significance level, for example, * means *p* < 0.05, ** means *p* < 0.01, and *** means *p* < 0.001. NS not significant

If the hypothesis that SNS overactivation is sufficient to impair corneal wound healing were true, we could predict that exogenously administered CChs would exaggerate wound-induced inflammation. Indeed, a local bolus of a nonselective β-AR agonist, isoepinephrine (an isopropylaminomethyl analog of epinephrine; 0.2 molL^−1^, 5 µL per eye every 6 h), administered immediately after corneal abrasion recapitulated many of the enhanced responses seen after ATSE. Mice exposed to these sustained isoepinephrine levels exhibited significantly retarded wound closure (Fig. 3a (L3), b) and decreased proliferative ability (Fig. 3c) compared to saline-treated control mice. To investigate how increased localized levels of isoepinephrine influence the dynamics of neutrophil and γδ T cell infiltration within the wounded cornea, we counted anti-Ly6G-FITC-labeled cells in four wound center fields and anti-GL3-PE-labeled cells from limbus to limbus in nine total fields (Field 4 in Supplementary Figure 4). At 12, 18, and 24 h after wounding, neutrophil recruitment was significantly augmented in the wounds of mice treated locally with isoepinephrine, in contrast to the gradual decline in the neutrophil number in wounded control corneas (Fig. 3d). At the same time points, the number of γδ T cells trafficked to the wounded cornea in isoepinephrine-treated mice was significantly higher than in saline-treated control mice and the ATSE-only group (Fig. 3e). Furthermore, we analyzed the transcription of three proinflammatory molecules, *NF-κB*, *IL-6*, and *IL-17A*, in whole-corneal mRNA using real-time quantitative reverse transcription PCR (qRT-PCR). The three molecules’ expression (Fig. 3f–h) was significantly higher in isoepinephrine-treated animals than RA animals. These data suggest that locally increased epinephrine concentration, as observed after ATSE, is sufficient to impair wound healing and exacerbate inflammation.

### SNS denervation and AR signaling rescue delayed wound repair

**Fig. 4:**
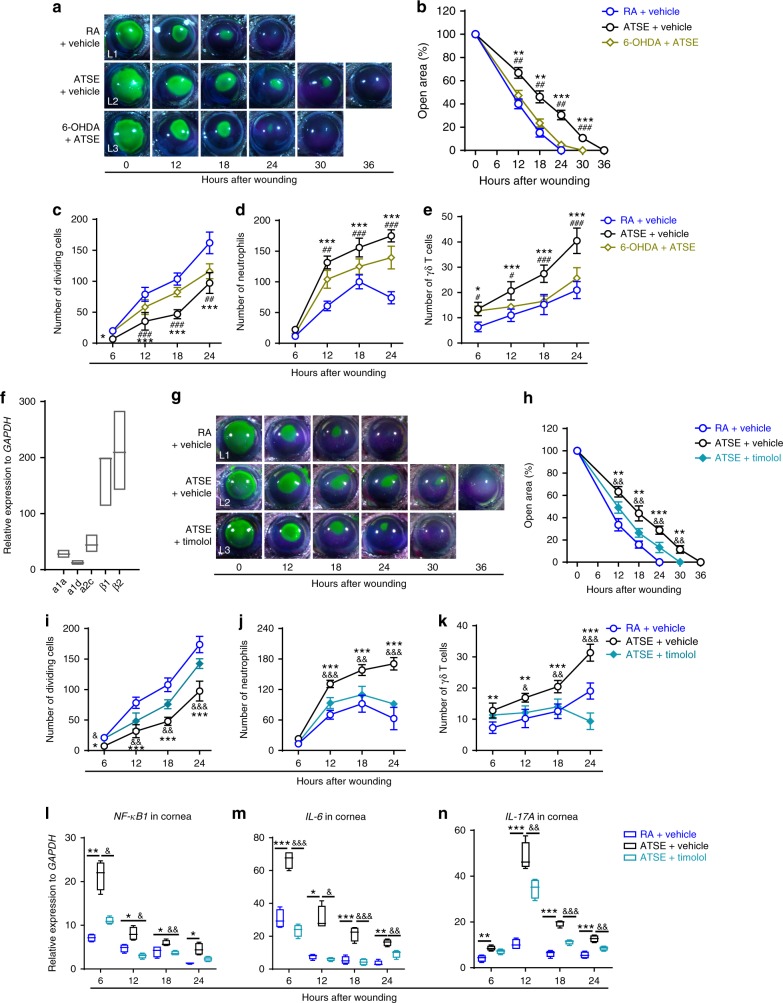
SNS denervation alleviates ATSE-induced corneal wound healing impairment, as does topical β-adrenergic receptor (AR) antagonist administration. **a** Representative open corneal wounds (revealed by topical fluorescein) and visible wound closure over time post wounding in RA and ATSE mice that received 6-OHDA or PBS (vehicle) pretreatments. **b** Percent decrease in open wound area over time after corneal abrasion in RA or ATSE mice pretreated with 6-OHDA or vehicle (two-way RM ANOVA, interaction *p* < 0.0001, Bonferroni’s multiple comparisons test, *n* = 6 corneas per time point per group). **c** Number of dividing epithelial cells over time post abrasion in RA or ATSE mice with 6-OHDA or vehicle pretreatment (two-way ANOVA, interaction *p* < 0.0001, Sidak’s multiple comparisons test, *n* = 6 corneas per time point per group). **d** Neutrophil influx into cornea over time post-abrasion in RA or ATSE mice after 6-OHDA or vehicle pretreatment (two-way ANOVA, interaction *p* < 0.0001, Sidak’s multiple comparisons test, *n* = 6 corneas per time point per group). **e** γδ T cell influx into limbus post abrasion in RA or ATSE mice pretreated with 6-OHDA or vehicle (two-way ANOVA, interaction *p* = 0.00012, Sidak’s multiple comparisons test, *n* = 6 corneas per time point per group). **f** Relative expression of adrenergic receptor mRNAs in flow-gating-sorted neutrophils as CD45^+^CD11b^+^Ly6G^+^ from 20 pooled whole wounded corneas 18 h after injury (Supplementary Figure 5). **g** Representative open corneal wounds (revealed by topical fluorescein) and visible wound closure over time (*n* = 6 corneas per time point per group). **h** Similar to **b** (two-way RM AVOVA, interaction *p* < 0.0001, Bonferroni’s multiple comparisons test, *n* = 6 corneas per time point per group). **i**–**k**) Similar to **c**, and **d**, **e**, respectively (two-way ANOVA, interaction *p* < 0.0001, Sidak’s multiple comparisons test, *n* = 6 corneas per time point per group) counted in eight limbus fields, ×40 magnification (Supplementary Figure 4); **l**–**n** relative *NF-kB*, *IL-6*, and *IL-17A* mRNA expression in whole wounded cornea of the RA, ATSE, and ATSE + timolol groups at 6, 12, 18, and 24 h after abrasion (one-way ANOVA, Tukey's post hoc, RA vs. TSE *p < 0.05, **p < 0.01, ***p < 0.001; RA vs. ATSE + timolol, ^##^p < 0.01, ^###^*p* < 0.001, *n* = 4) measured by qRT-PCR. Data represent three independent experiments. Symbols denoting statistical significance in **c**–**f**: *comparison between ATSE and RA groups; ^#^comparison between ATSE and ATSE + 6-OHDA with ^#^*p* < 0.05, ^##^*p* < 0.01, ^###^*p* < 0.001. ^&^ compares ATSE and ATSE + timolol groups. In **g**–**i**, symbols compare groups as indicated in figure. *^, #, &^*p* < 0.05, **^, ##, &&^*p* < 0.01; ***^, &&&, ###^*p* < 0.001

Given the regulation of the SNS and β-AR signaling in inflammation and corneal reepithelialization after corneal injury^57,58^, we examined the role of the SNS and its overactivation in ATSE-driven exacerbated inflammation and delayed corneal wound healing. We treated mice with 6-OHDA three times prior to corneal abrasion. Three days after the final 6-OHDA treatment, we mechanically wounded the corneal epithelium with our previously described protocol^5^. Subsequently, we monitored reepithelialization, epithelial cell division, and the influx of neutrophils and γδ T cells into the wounded area at 6-h intervals after corneal abrasion. Remarkably, the rate of reepithelialization was significantly accelerated in 6-OHDA-pretreated mice at 12, 18, and 24 h after abrasion compared to those in the ATSE-only group, as shown by fluorescein labeling (Fig. 4a, b). The number of mitotic epithelial cells in 6-OHDA-pretreated mice was significantly higher (Fig. 4c) at 12, 18, and 24 h post-corneal abrasion compared to the ATSE-only treatment group. However, the number of mitotic epithelial cells in the 6-OHDA + ATSE group was still lower than in the RA group. The number of neutrophils and γδ T cells trafficked to the wound in the 6-OHDA + ATSE treatment group was significantly lower at 6, 12, 18, and 24 h post-corneal abrasion compared to the ATSE-only treatment group (Fig. 4d, e). However, the number of these immune cells in the 6-OHDA + ATSE treatment group were still higher than in the RA group (Fig. 4d, e). These results suggest that impairment induced by ATSE treatment can be partially improved by SNS denervation.

The physiological effects of the SNS are mediated by CChs being released from nerve endings and binding to α-ARs and/or β-ARs, which are variably expressed in different immune cells^36^. Data from the previous section of our study showed that neutrophils are the major inflammatory cells that ATSE stimulates in response to excessive inflammatory response. Chemical sympathectomy can significantly reduce the infiltration of a large number of neutrophils into the traumatized cornea after ATSE treatment. To determine the molecular mechanism by which sympathetic nerves regulate neutrophil infiltration, we first used qRT-PCR to identify the expression profiles of ARs in purified neutrophils from wounded corneas 18 h after wounding (isolation of neutrophils shown in Supplementary Figure 5). The neutrophils predominantly expressed β1- and β2-ARs (Fig. 4f). To observe the role of these β-ARs in the dramatic ATSE-induced inflammatory response, we treated mice with the nonselective β-AR antagonist timolol immediately after smoke exposure and corneal abrasion and then measured the rate of wound closure, epithelial cell division, and neutrophil infiltration into the wounded corneas. Treatment with timolol significantly promoted wound closure (Fig. 4g (L2, L3), h) and increased the amount of cell division (Fig. 4i). Simultaneously, neutrophil and γδ T cell trafficking to the wounded cornea was significantly reduced (Fig. 4j, k). Finally, we observed the effect of topical timolol administration on *NF-κB*, *IL-6*, and *IL-17A* expression in whole-corneal mRNA after ATSE by qRT-PCR. Topical timolol administration significantly suppressed the enhanced expression of the three proinflammatory molecules induced by ATSE (Fig. 4l–n), suggesting that blocking β-AR signaling reduced impairment of wound healing in ATSE-treated mice.

### ATSE activates the NF-κB signaling pathway via the SNS

**Fig. 5:**
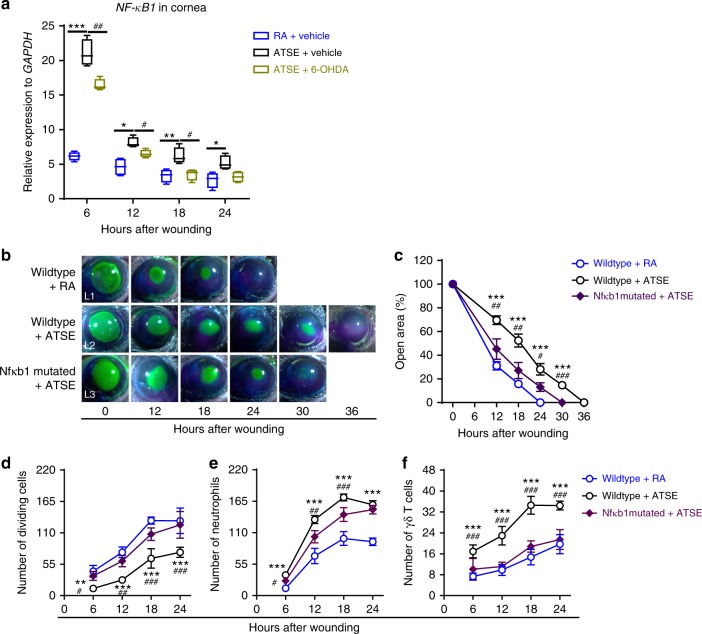
ATSE-induced impairment of corneal wound healing requires NF-κB signaling. **a** Relative *NF-κB* expression using mRNA isolated from whole wounded corneas of the RA, ATSE, and ATSE + 6-OHDA groups at 6, 12, 18, and 24 h after corneal abrasion (one-way ANOVA, Tukey's post hoc test, RA vs. ATSE, *p < 0.05, **p < 0.01, ***p < 0.001; RA vs. ATSE + 6-OHDA, ^##^*p* < 0.01, ^###^*p* < 0.001, *n* = 4). **b** Representative open corneal wounds (revealed by topical fluorescein) and visible wound closure over time post wounding. **c** Percent decrease in open wound area over time after wounding (two-way RM ANOVA, interaction *p* = 0.00002, Bonferroni’s multiple comparisons test, *n* = 6 corneas per time point per group). **d** Dividing epithelial cells over time post-wounding (two-way ANOVA, interaction *p* = 0.0278, Sidak’s multiple comparisons test, *n* = 6 corneas per time point per group). **e** Neutrophil influx into the cornea over time after corneal abrasion (two-way AVOVA, interaction *p* < 0.0001, Sidak’s multiple comparisons test, *n* = 6 corneas per time point per group). **f** γδ T cell influx into wounded corneas over time (two-way ANOVA, interaction *p* = 0.0020, Sidak’s multiple comparisons test, *n* = 6 corneas per time point per group). * comparison between ATSE and RA treatments in wild-type mice; ^#^ comparison between ATSE-treated wild-type mice and ATSE + *Nfkb1*-mutated animals. *^, #^ denote *p* < 0.05, **^, ##^*p* < 0.01, and ***^, ###^*p* < 0.001, comparing groups as indicated

A key role of the NF-κB family is the regulation of a large array of genes involved in different immune and inflammatory responses^59^. Therefore, we predicted that SNS overactivation in ATSE-treated mice could trigger neutrophils to release NF-κB. We first measured *NF-κB* gene expression in the wounded cornea after ATSE. In the RA group, *NF-κB* expression peaked at 6 h after injury and then declined. In the ATSE-treated group, *NF-κB* expression was constantly higher and lasted for at least 24 h after injury (Fig. 5a). To further validate the effect of NF-κB, we subjected *Nfκb1* mutant mice to ATSE and abrasion. The *Nfkb1-*mutated + ATSE group’s corneal epithelial wounds were completely closed at 30 h (Fig. 5b (L3), c); the wild-type + ATSE group’s wounds closed only after 36 h (Fig. 5b (L2), c). At all the time points measured post injury, the number of dividing cells in the *Nfkb*1-mutated, ATSE-treated mouse corneas was significantly higher than in the wild-type mice that received ATSE treatment (Fig. 5d). Additionally, the influx of neutrophils and γδ T cells into the wounded cornea in *Nfκb1-*mutated mice after ATSE was significantly lower than in the wild-type ATSE groups (Fig. 5e, f). Together, these data indicate that NF-κB1 inactivation can effectively improve ATSE-impaired healing.

**Fig. 6:**
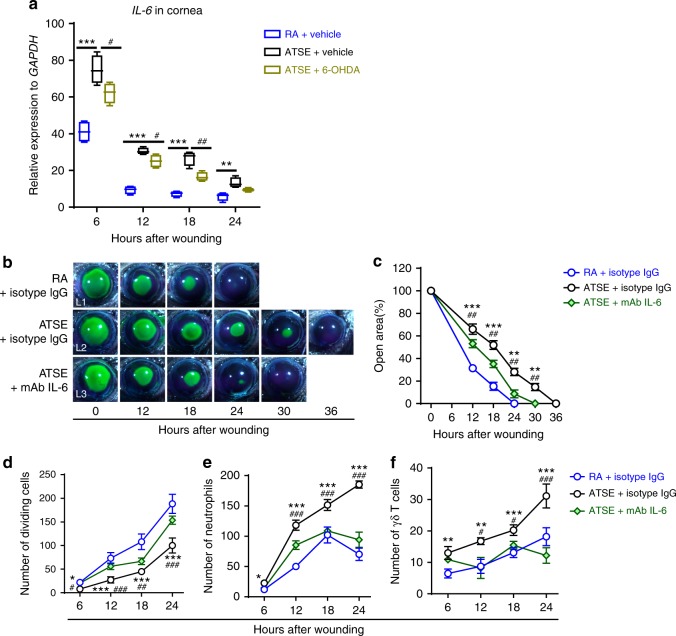
Effects of IL-6 neutralization (using IL-6 antibody mAb IL-6) on ATSE-induced wound healing delay and exacerbated inflammation. **a** Relative expression of *IL-6* using mRNA isolated from whole wounded corneas of RA, ATSE, and ATSE + 6-OHDA mice at 6, 12, 18, and 24 h after corneal abrasion (one-way ANOVA, Tukey's post hoc test, RA vs. ATSE, *p < 0.05, **p < 0.01, ***p < 0.001; RA vs. ATSE + 6-OHDA, ^##^*p* < 0.01, ^###^*p* < 0.001, *n* = 4 corneas per time point per group). Data represent three independent experiments. **b** Representative open corneal wounds (revealed by topical fluorescein) and visible wound closure over time post abrasion. **c** Percent decrease in open wound area over time after corneal wounding of RA or ATSE mice treated with mAb IL-6 or isotype IgG as a control (two-way RM ANOVA, interaction *p* < 0.0001, Bonferroni’s multiple comparisons test, *n* = 6 corneas per time point per group). **d** Number of dividing epithelial cells over time after wounding in RA or ATSE mice treated with mAb IL-6 or isotype IgG control (two-way ANOVA, interaction *p* < 0.0001, Sidak’s multiple comparisons test, *n* = 6 corneas per time point per group). **e** Neutrophil influx into the cornea over time after corneal abrasion in RA or ATSE mice treated with mAb IL-6 or isotype IgG control (two-way ANOVA, interaction *p* < 0.0001, Sidak’s multiple comparisons test, *n* = 6 corneas per time point per group). **f** γδ T cell influx into injured cornea over time after corneal abrasion in RA or ATSE mice treated with mAb IL-6 or isotype IgG control (two-way ANOVA, interaction *p* < 0.0001, Sidak’s multiple comparisons test, *n* = 6 corneas per time point per group). *comparison between ATSE + isotype IgG and RA + isotype IgG treatment groups with *p < 0.05, **p < 0.01, and ***p < 0.001; ^#^ comparison between ATSE + isotype IgG and ATSE + mAb IL-6 groups with ^#^*p* < 0.05, ^##^*p* < 0.01, and ^###^*p* < 0.001

Since the SNS activates NF-κB signaling in immune cells by completely unknown mechanisms^41,60,61^, we hypothesized that ATSE enhances the NF-κB pathway by increasing SNS neural activity. To assess the effect of SNS denervation and activation on NF-κB expression in injured corneas after ATSE, we sympathectomized the mice with 6-OHDA and detected NF-κB mRNA expression in injured corneas after ATSE. The NF-κB expression level after abrasion in the 6-OHDA-pretreated group was significantly lower than in the ATSE-only group (Fig. 6a), suggesting that increased SNS signaling activity can enhance *NF-κB* expression in the murine cornea.

### SNS denervation and the AR blockade inhibit IL-6 production

IL-6 plays a major role in the regulation of corneal inflammation and wound healing^62–64^. Therefore, we determined the effect of IL-6 on ATSE-induced exacerbated inflammation and delayed reepithelialization. First, we detected *IL-6* mRNA expression after abrasion using qRT-PCR. *IL-6* expression peaked 6 h after injury in the RA animals. However, in the smoke-exposed animals, *IL-6* expression dramatically increased at 6, 12, 18, and 24 h after injury compared to RA mice (Fig. 6a). To examine the SNS’s role in this increase, we chemically ablated SNS fibers with 6-OHDA, and then subjected the animals to ATSE and corneal abrasion. *IL-6* expression in the injured cornea from 6-OHDA + ATSE-treated animals was significantly lower than in the RA and ATSE groups at 6, 12, and 18 h after corneal abrasion (Fig. 6a). We also determined the role of the β2-AR signaling pathway in enhancing *IL-6* expression after ATSE by topically applying timolol to the ocular surface immediately after smoke exposure. The *IL-6* expression level in abraded corneas was significantly lower in the β2 blocker + ATSE animals than in the RA and ATSE-only groups (Fig. 4m). This suggests that increased SNS signaling and activity leads to a further increase in *IL-6* expression after ATSE via the β2-AR signaling pathway.

To validate the role of IL-6 in ATSE-impaired healing, we locally administered an anti-IL-6 antibody to neutralize the effect of endogenous IL-6^63,65,66^ and then compared corneal healing between groups. Local administration of the anti-IL-6 antibody in the ATSE group markedly improved delayed reepithelialization (Fig. 6b (L2, L3), c) and epithelial cell division (Fig. 6d) and decreased neutrophil and γδ T cell trafficking to the injured cornea (Fig. 6e, f) compared to the ATSE group treated with isotype immunoglobulin G (IgG) (control). Thus, post-wounding neutralization of IL-6 has a potential therapeutic effect in reversing post-ATSE healing impairment.

### SNS denervation and AR blockade inhibit IL-17A production

**Fig. 7:**
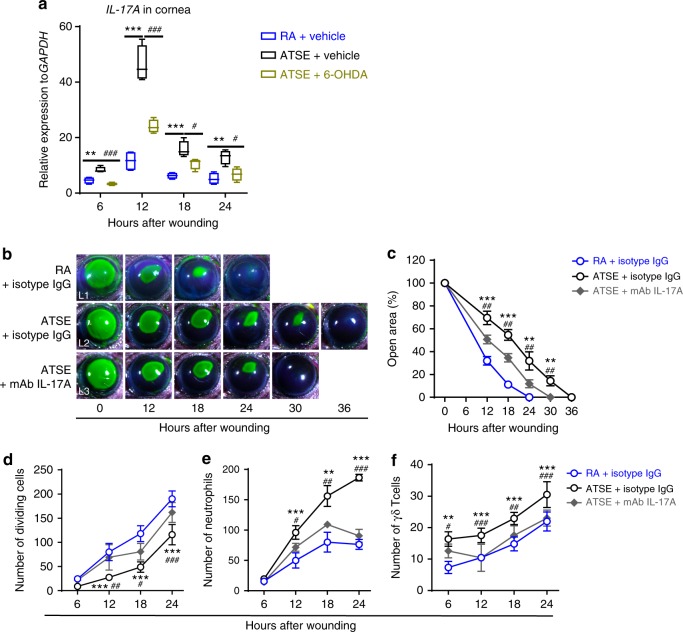
IL-17A is a critical mediator of ATSE-induced delayed wound healing and exacerbated inflammation. **a** Relative *IL-17A* expression using mRNA isolated from whole wounded corneas of the RA, ATSE, and ATSE + 6-OHDA groups at 6, 12, 18, and 24 h post-corneal abrasion (one-way ANOVA, Tukey's post hoc test, RA vs. ATSE, *p < 0.05, **p < 0.01, ***p < 0.001; RA vs. ATSE + 6-OHDA, ^##^*p* < 0.01, ^###^*p* < 0.001, *n* = 4 corneas per time point per group). The data represent three independent experiments. **b** Representative open corneal wounds (revealed by topical fluorescein) and visible wound closure over time. **c** Percent decrease in open wound area over time post-wounding (two-way RM ANOVA, interaction *p* < 0.0001, Bonferroni’s multiple comparisons test, *n* = 6 corneas per time point per group). **d** Number of dividing epithelial cells over time post-wounding (two-way AVOVA, interaction *p* = 0.00047, Sidak’s multiple comparisons test, *n* = 6 corneas per time point per group). **e** Neutrophil influx into the cornea over time after corneal abrasion (two-way ANOVA, interaction *p* < 0.0001, Sidak’s multiple comparisons test, *n* = 6 corneas per time point per group). **f** γδ T cell influx into the cornea over time after corneal abrasion (two-way ANOVA, interaction *p* = 0.31888, Sidak’s multiple comparisons test, *n* = 6 corneas per time point per group). *comparison between the ATSE + isotype IgG and RA + isotype IgG groups with *p < 0.05, **p < 0.01, and ***p < 0.001; ^#^comparison between the ATSE + isotype IgG and ATSE + mAb IL-17A groups with ^#^*p* < 0.05, ^##^*p* < 0.01, and ^###^*p* < 0.001

IL-17A is the primary coordinator of the initial and adaptive immune responses and a major chemokine that recruits and mobilizes neutrophils during inflammatory response^67^. To determine whether increased SNS signaling activity after ATSE affected IL-17A production in the cornea, we repeated the experiments conducted above. We detected post-abrasion *IL-17A* expression in the cornea using qRT-PCR. *IL-17A* expression peaked at 12 h in the RA group after corneal injury, whereas ATSE treatment significantly increased *IL-17A* expression compared to the RA group at 6, 12, 18, and 24 h post abrasion (Fig. 7a). However, corneal *IL-17A* expression was significantly lower in the 6-OHDA + ATSE group than in the ATSE group (Fig. 7a), suggesting that SNS denervation inhibits local IL-17A production after ATSE. Moreover, we validated the role of IL-17 in ATSE-induced abnormal wound healing. Anti-IL-17 antibody was locally administered to neutralize endogenous IL-17^35^. We also compared corneal healing between the groups and found that local anti-IL-17 antibody administration in the ATSE group resulted in a significant recovery of delayed reepithelialization (Fig. 7b (L2, L3), c), increased epithelial cell division (Fig. 7d), and decreased neutrophil (Fig. 7e) and γδ T cell trafficking (Fig. 7f) to the injured cornea compared to the isotype IgG control group. These data suggest that post-wounding neutralization of IL-17A, like IL-6, could have a therapeutic effect in reversing the impairment of post-ATSE wound healing.

Given the different sources of IL-17A—a variety of immune cells such as γδ T cells, T-helper type (Th17) cells, natural killer (NK) cells, and NKT cells^63^—we wanted to identify the primary IL-17A source in the post-abrasion cornea. We used intracellular IL-17A staining and immunocytotyping on single-corneal cells 18 h post trauma. As predicted, flow cytometry showed that all the IL-17A^+^ cells were from the CD45^+^ leukocyte population (Supplementary Figure 6A). Based on three independent experiments, our data showed that ~41.22 ± 0.47% of these IL-17A^+^ cells were Ly6G-positive neutrophils, ~5.32 ± 0.64% of them were GL3^+^ γδ T cells, and **~**53.46 ± 0.28% of them were CD45^+^ leukocytes of unknown phenotype (Supplementary Figure 6B and C). These results suggest that post-corneal-injury local IL-17A production is mainly derived from CD45^+^ leukocytes, including γδ T cells and neutrophils.

### α1-AR blockage expressed on γδ T cells rescues wound repair

**Fig. 8:**
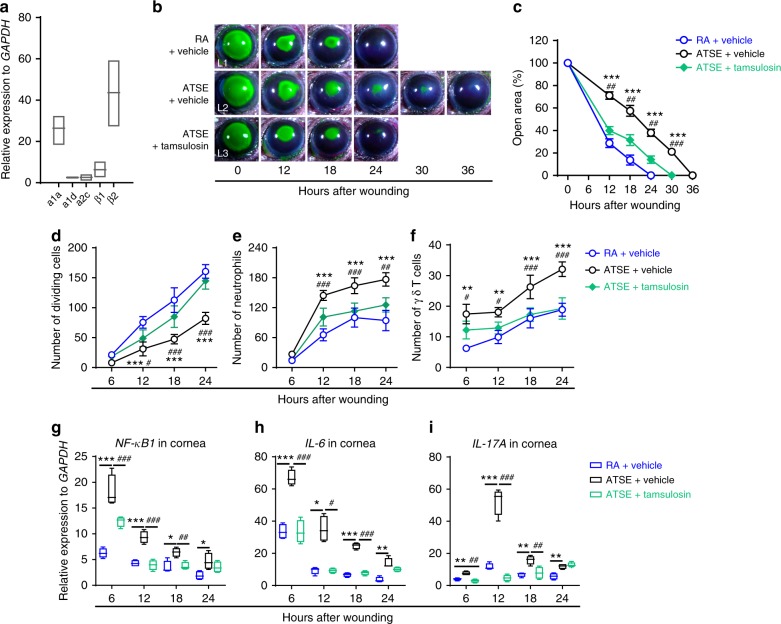
The α1a-AR antagonist tamsulosin alleviates delayed wound healing and exacerbated inflammation. **a** Relative expression of adrenergic receptors in sorted CD45^+^GL3^+^ γδ T cells from 20 pooled wounded corneas 18 h after corneal abrasion (Supplementary Figure 5). **b** Representative open corneal wounds (revealed by topical fluorescein) and visible wound closure over time post wounding. **c** Percent decrease in open wound area over time after wounding in the RA, ATSE, or ATSE + tamsulosin-treated mice (two-way RM ANOVA, interaction *p* < 0.0001, Bonferroni’s multiple comparisons test, *n* = 6 corneas per time point per group). **d** Epithelial cell division over time after wounding in the RA, ATSE, and ATSE + tamsulosin groups (two-way ANOVA, interaction *p* < 0.0001, Sidak’s multiple comparisons test, *n* = 6 corneas per time point per group). **e** Neutrophil influx into the cornea over time after injury in RA, ATSE, and ATSE + tamsulosin groups (two-way ANOVA, interaction *p* < 0.0001, Sidak’s multiple comparisons test, *n* = 6 corneas per time point per group). **f** γδ T cell influx into the wounded cornea over time after corneal abrasion in the RA, ATSE, and ATSE + tamsulosin groups (two-way ANOVA, interaction *p* = 0.00513, Sidak’s multiple comparisons test, *n* = 6 corneas per time point per group). **g–i** Whole-cornea mRNA expression of *NF-kB*, *IL-6*, and *IL-17A* in injured corneas of the RA, ATSE, and ATSE + topical tamsulosin-treated groups at 6, 12, 18, and 24 h after abrasion (one-way ANOVA, Tukey's post hoc test, RA vs. ATSE, *p < 0.05, **p < 0.01, ***p < 0.001, RA vs. ATSE + tamsulosin; ^#^*p* < 0.05, ^##^*p* < 0.01, ^###^*p* < 0.001, *n* = 6 corneas per group). Symbols denoting statistical significance in **c**–**f**: * compares the RA and ATSE groups; ^#^ compares the ATSE and ATSE + tamsulosin groups. In **g**–**i**, symbols compare groups as indicated. *^,^^#^*p* < 0.05; **^, ##^*p* < 0.01; ***^, ###^*p* < 0.001

SNS stress and infusions of epinephrine and norepinephrine cause rapid and transient mobilization of γδ T cells into circulation^53,54^. Therefore, we sought to link the roles of ARs expressed by γδ T cells to these cells’ influx into the cornea. First, we analyzed the AR transcriptional profile in infiltrated γδ T cells that were sorted and purified from wounded corneas 18 h post abrasion (Supplementary Figure 5). These cells preferentially expressed β2- and α1a-AR receptors (Fig. 8a). Preferential β2 receptor expression was consistent with previous functional observations and data (Fig. 4f); topical β-blockers promote reepithelialization, increase epithelial cell division, and inhibit inflammation (including neutrophil and γδ T cell recruitment and proinflammatory cytokine expression) in injured corneas. To validate the effect of α1a receptor expression on corneal γδ T cells, either an α1a-AR agonist or antagonist was topically administered immediately post wounding. Topical administration of the selective α1-AR antagonist tamsulosin significantly promoted reepithelialization (Fig. 8b (L2, L3), c) and epithelial cell division (Fig. 8d), inhibited neutrophil (Fig. 8e) and γδ T cell influx (Fig. 8f) into the wound, and inhibited proinflammatory cytokine expression (Fig. 8g–i) in ATSE-treated wounded corneas. Altogether, this indicates that γδ T cells express α1-ARs and β2-ARs. Additionally, our findings reveal the importance of α1-ARs expressed on γδ T cells for initiating the cells’ infiltration after ATSE and/or corneal wounding.

### Interaction among IL-6, IL-17A, and NF-κB1 after injury

**Fig. 9:**
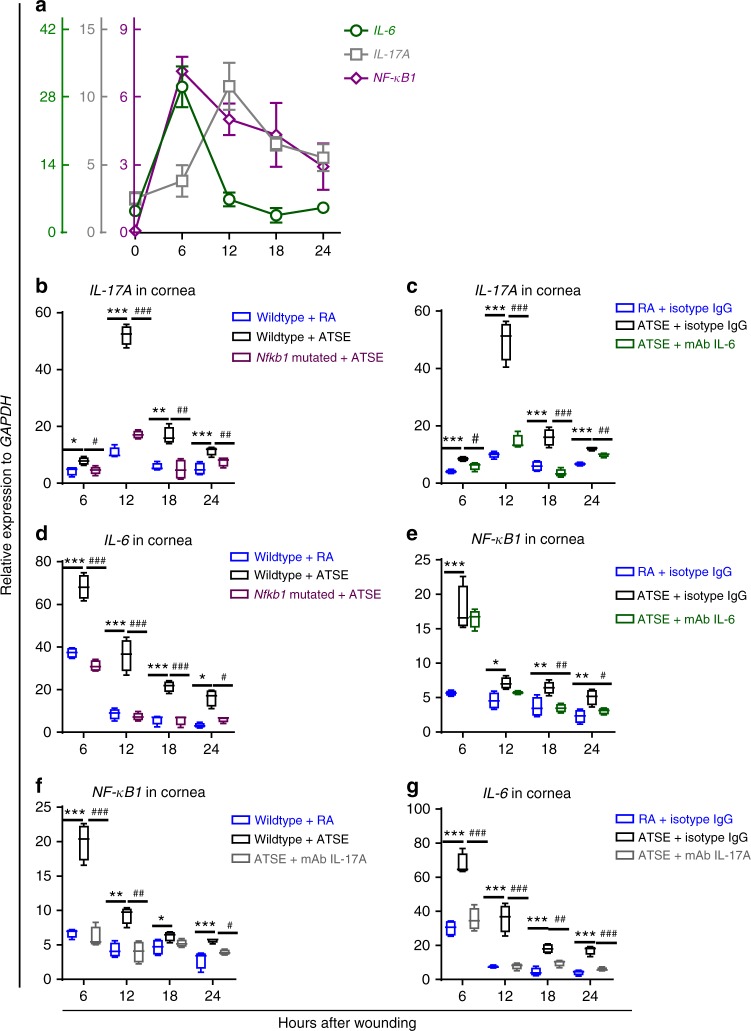
Interaction and feedback among IL-6, IL-17A, and NF-κB after ATSE. **a** Kinetics of the relative expression of *NF-κB*, *IL-17A*, and *IL-6* using mRNA isolated from whole wounded corneas in the RA group at 6, 12, 18, and 24 h after corneal abrasion. **b** Effects of genetic NF-κB deactivation on the expression of *IL-17A* at different time points after corneal abrasion in the RA and ATSE groups. **c** Effects of local IL-6 neutralization (with IL-6 antibody mAb IL-6) on the expression of *IL-17A* at different time points after corneal abrasion in the RA and ATSE groups. **d** Effects of NF-κB genetic deactivation on the local expression of *IL-6* at different time points after corneal abrasion in the RA and ATSE groups. **e** Effects of local IL-6 neutralization (with mAb IL-6) on the local expression of *NF-κB* at different time points after corneal abrasion in the RA and ATSE groups. **f** Effects of local IL-17A neutralization on the mRNA expression of whole-corneal *NF-κB1* at different time points after corneal abrasion in the RA and ATSE groups after wounding. **g** Effects of local IL-17A neutralization (using IL-17A antibody mAb IL-17A) on the gene expression of *IL-6* in the whole cornea at different time points after corneal abrasion in the RA and ATSE groups. *n* = 4 corneas per time point in each group. The data represent three independent experiments. One-way ANOVA, Tukey's post hoc test. *ATSE vs. RA; ^#^ATSE vs. ATSE + *Nfkb1*-mutated animals (in **b**, **d**), ATSE + mAb IL-6 (in **c**, **e**), and ATSE + mAb IL-17A (in **f**, **g**). *^, #^*p* < 0.05; **^, ##^*p* < 0.01; ***^, ###^*p* < 0.001

Our results showed that NF-κB1, IL-6, and IL-17A play different and important roles in exacerbated inflammatory response to post-ATSE injury. However, the spatial and temporal relationships among the three cytokines are still unclear. To confirm their relationship, we compared their transcription level kinetics in RA mice post-corneal abrasion. *NF-κB1* and *IL-6* expression peaked 6 h after wounding; *IL-17A* expression peaked 12 h after (Fig. 9a). Moreover, *IL-17A* expression in *Nfkb1*-mutated mice and in wild-type mice after topical neutralization of IL-6 was markedly reduced (Fig. 9b, c), suggesting that NF-κB1 and IL-6 are located upstream of IL-17A production in the inflammatory signaling pathway. To explore the reciprocal relationship between NF-κB1 and IL-6, we used genetic (*Nfkb1*-mutated animals) and chemical (topical IL-6 neutralization antibodies) techniques to show how the molecules influence each other’s transcription. Genetic NF-κB1 deactivation significantly reduced *IL-6* expression after the RA and ATSE treatments (Fig. 9d). However, IL-6 neutralization antibodies did not interfere with NF-κB1 expression 6 h after wounding, although they did significantly reduce NF-κB1 expression at 12, 18, and 24 h post injury (Fig. 9e). Consistent with the classical hypothesis, these data reveal a unique upstream location of NF-κB1 in the initiation of the inflammatory response to wounding. We also performed reciprocal experiments on IL-17A vs. NF-κB1 and IL-17A vs. IL-6. Anti-IL-17A neutralization antibodies suppressed transcription of both *NF-κB1* and *IL-6* (Fig. 9f, g), indicating that downstream IL-17A can regulate its partners upstream. Altogether, these findings suggest reciprocal, positive relationships between NF-κB1 and IL-17A and between IL-17A and IL-6; there is a positive relationship for NF-κB1 to IL-6 but not vice versa, at least at 6 h. IL-17A might play a unique, pivotal role in amplifying and maintaining inflammatory wound response via the feedback loop stimulating IL-6 production and the functional change of NF-κB1 after wounding plus ATSE.

## Discussion

We describe here an important mechanism linking ATSE to SNS activation and the consequent release of CChs. We show that ATSE-induced CChs can activate the NF-κB signaling pathway, augment IL-6 and IL-17A release, and mobilize leukocytes from the bone marrow into the circulation and then to the corneal limbus through AR action. Post-corneal abrasion, the newly trafficked leukocytes that express ARs relay the CCh stimulation signals from the SNS to the wounded cornea. The consequent enhancement of *IL-6* and *IL-17A* expression recruits more inflammatory cells to the wound site, leading to ATSE-triggered impairment of wound healing. These experiments highlight the impressive activation of the SNS signaling pathway in response to ATSE in the pathogenesis of ATSE-induced inflammation and delayed healing.

Corneal healing after mechanical abrasion is influenced by extrinsic factors such as stress, medication, disrupted circadian rhythms^68^, and diabetes^69^. Our study demonstrates that tobacco smoke also alters wound healing by inducing SNS overactivation. SNS innervate almost all organs and tissues, including mammalian corneas^30,31^. The SNS can directly regulate inflammatory reactions in lymphoid and nonlymphoid organs through AR function^35^. In healthy subjects, infusion of the nonselective β-AR agonist isoproterenol at physiological concentrations or increased concentration of epinephrine caused by psychological stress can rapidly and transiently mobilize a variety of immune cells into circulation in a dose-dependent manner^53,54^. Our study reveals that enhanced SNS tone or activity triggered by ATSE can also cause similar leukocytosis in injured and uninjured corneas. These data are consistent with previous human studies^55,56^.

Although chemical SNS denervation can correct excessive inflammatory reactions and restore reepithelialization, the molecular mechanism linking SNS activation and inflammatory cell infiltration to the injured cornea had not been previously elucidated. We show that infiltrated neutrophils preferentially express β1-AR and β2-AR, and a pharmacological β-AR blockade significantly improves wound healing. Additionally, we reveal for the first time, to our knowledge, that γδ T cell trafficking to the injured cornea is controlled by β2-ARs and α1a-ARs. Together, all this information indicates that decreasing or blocking SNS activation may be therapeutic for corneal wound healing after ATSE.

Injury-induced inflammation can promote or hinder wound healing and tissue regeneration. Neutrophils are the first immune cells recruited to wound sites; on the one hand, the enzymes and antimicrobial peptides produced by these cells scavenge apoptotic bodies and cell debris and inhibit pathogen growth^49^. On the other hand, excessive neutrophil infiltration and enzyme and ROS production after tissue injury can be toxic to healing and can inhibit wound repair^5,70^. We find that inhibiting neutrophil influx with a chemokine receptor antagonist mitigates ATSE-induced impairments of corneal reepithelialization and epithelial cell division. However, the CXCR2 antagonist also uses reduced neutrophil numbers well below those of even the RA group. Given that some level of neutrophil influx is the normal immune response required for wound repair, any such therapeutic intervention would need to be carefully titrated to maintain a specific neutrophil level range and not completely undermine the body’s healing ability.

The γδ T cell also contributes to corneal healing through local production of epithelial growth factors (e.g., fibroblast growth factor 7) and inflammatory cytokines (e.g., IL-17A and IL-22)^3,4,71^. In γδ T cell-deficient mice, corneal responses to abrasion are decreased inflammatory response and delayed repair^3^. Topical IL-17A can largely alleviate delays^3,4^. Consistent with previous work, corneas attract more γδ T cells and increase *IL-17A* transcription post ATSE. IL-17A was once considered to be produced only by Th17 cells and γδ T cells^3,4,6^, but recent studies have shown that various immune cells, including neutrophils^57^, can produce IL-17A. We show that only half the IL-17A^+^ cells were recruited to the injured cornea from γδ T cells and neutrophils. The other half came from GL3^−^Ly6G^−^CD45^+^ leukocytes. Recent studies have also found that ocular surface IL-17A^+^ cells are derived from other heterogeneous leukocyte populations, including innate-like αβ T cells and innate lymphoid T cells in addition to γδ T cells^72,73^. More importantly, we find that topical anti-IL-17A administration greatly improves damage and accelerates healing. All of this suggests that IL-17A-producing leukocytes are key in exacerbating inflammation and delaying injury repair in a smoke-exposed environment. However, the phenotype and characteristics of IL-17A-producing leukocytes that are not γδ T cells and neutrophils need further investigation.

NF-κB is involved in ocular surface functions such as corneal development, anti-infection processes, epithelial barrier integrity, and wound healing^74,75^. Many external factors, such as unhealthy diet, circadian rhythm disruption, and sun/ultraviolet-ray exposure, lead to NF-κB activation^46,47^. ATSE is another environmental factor that can cause rapid and robust functional NF-κB changes in wounded corneas, consistent with the recent discovery that ATSE activates human airway lymphocytes and oral-cell NF-κB^76,77^. Functional NF-κB change is required for ATSE-associated impairment of corneal healing. Thus, our results suggest a new mechanism by which ATSE is important in corneal injury pathology through overstimulating the SNS to activate the NF-κB signaling pathway.

IL-6, a key multifunctional cytokine regulating inflammatory responses and immune reactions, amplifies inflammatory response by rapidly recruiting leukocytes to infected or injured sites. Corneal infection and trauma are often associated with significant IL-6 increases^78,79^. Additionally, IL-6 overproduction is pathologically involved in many corneal inflammatory problems such as herpes simplex viral-1 keratitis^65,66^ and corneal chemical burns^80^. Our study supports the importance of IL-6 in corneal injury-induced inflammation augmented by ATSE. Therefore, blocking IL-6 activity is one therapeutic option for these diseases.

Our ATSE experiments have some limitations that may make the results difficult to translate to the clinical domain and the human condition. First, the ATSE protocol used intense levels rarely observed in smokers. Second, since mice are obligatory nasal breathers, some toxic products that would normally be inhaled by mouth in humans may have been deposited in the mouse nasal passages, leading to effects that may not be replicated in other species. Third, the diverse toxins present in tobacco smoke, such as ROS, ROS inducers, carbon monoxide, hydrogen cyanide, and free radicals^81,82^, can enter cells and cause oxidative DNA damage and even cell death. Therefore, more refined future experiments should help distinguish the contributions and collaborative effects of the individual components of tobacco smoke in wound healing impairment. Although these restrictions must be considered when interpreting our results, our data clearly demonstrate that ATSE impairs corneal healing and exacerbates the inflammatory response. Thus, we highlight the necessity for patients with corneal injuries or surgery to avoid ATSE.

**Fig. 10:**
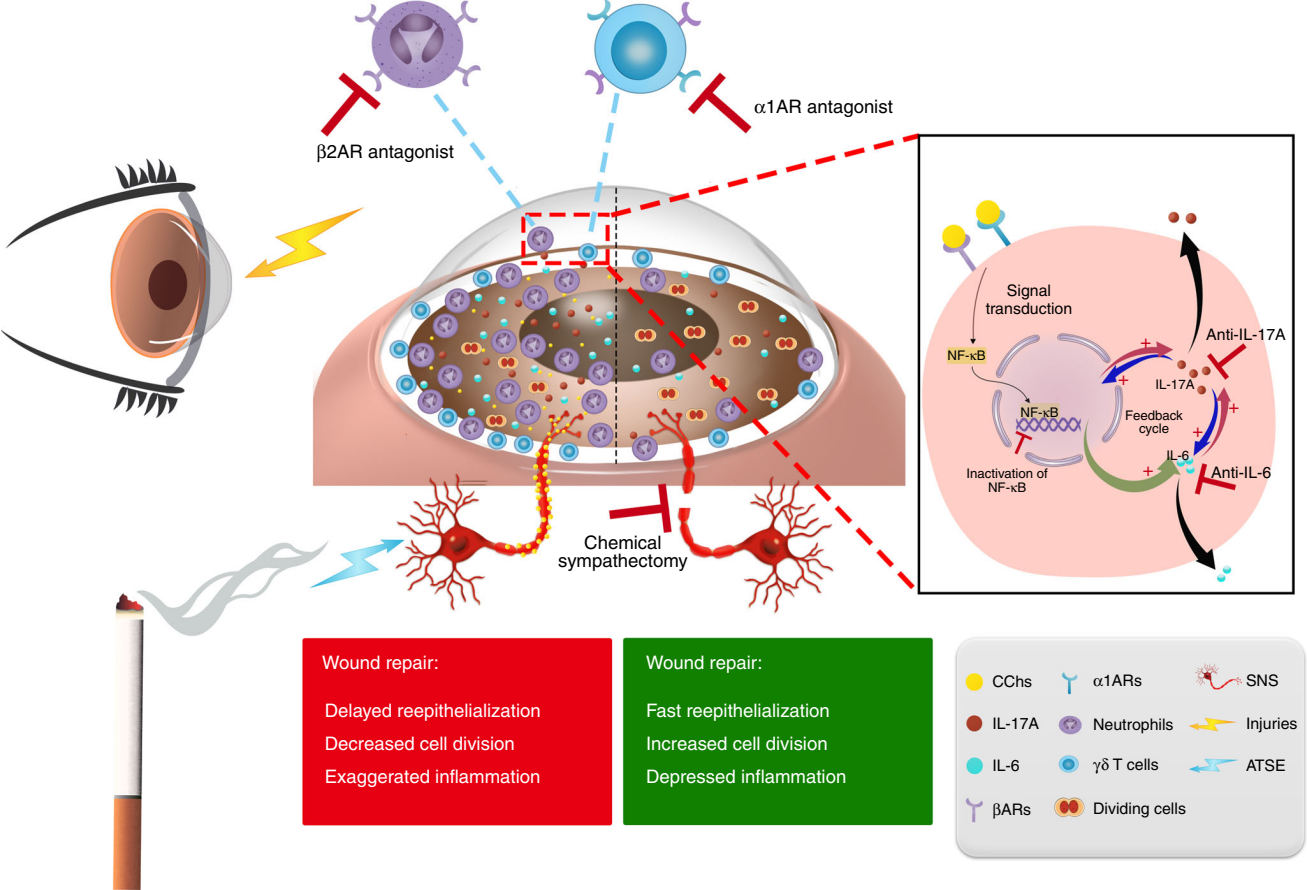
Hypothetical mechanism by which ATSE aggravates the inflammatory reaction and delays wound healing by triggering the overactivation of the SNS. Acute smoke exposure increases the activity of the SNS, which releases CChs from nerve endings. CChs stimulate the mobilization of different leukocytes from the bone marrow into the circulation and stimulate traffic to the corneal limbus. At the same time, CChs enhance the activation of the NF-κB signaling pathway by stimulating different ARs expressed on corneal and immune cells such as γδ T cells, neutrophils, and other leukocytes to produce inflammatory cytokines such as IL-6 and IL-17A. IL-17A also further amplifies the inflammatory reaction to injuries and impairs the wound healing process by a positive feedback loop with the production of IL-6 and the change of NF-κB1. This mechanism results in corneal wound healing impairments such as delayed reepithelialization, reduced cell division, and exacerbated inflammation. The red lines at the top of the schematic represent target sites along the mechanism where therapeutic intervention can reduce ATSE-induced healing impairments

In summary, we have discovered a new molecular link between SNS overactivation and corneal wound healing following ATSE (Fig. 10). Enhanced SNS activity induced by ATSE leads to a change in the NF-κB signaling pathway. Systemic CCh increase due to SNS overactivation augments the mobilization of leukocytes such as neutrophils and γδ T cells into the circulation, unwounded corneal limbi, and injured corneas. The exaggerated post-injury inflammatory response delays wound repair. This cascade is further amplified by cross-talk and positive feedback among NF-κB1, IL-6, and IL-17A. These data highlight the important regulatory role of the SNS on the ocular surface in response to environmental changes. This study provides a rationale for employing α-AR and β-AR antagonists to prevent certain ocular surface impairments in an acute tobacco smoke environment.

## Methods

### Animals

All animal experiments were performed in accordance with the Statement for the Use of Animals in Ophthalmic and Visual Research with approval from the Jinan University Animal Ethical Review Committee. C57BL/6J female mice were purchased from the Guangdong Medical Laboratory Animal Center (Foshan, China) at 10–14 weeks of age. *Nfkb1* gene-mutated (cysteine-alanine (Cys-Ala), CA) mice were procured from the Model Animal Research Center of Nanjing University (Nanjing, China) and bred in-house as homozygous lines on the C57BL/6J background. The *Nfkb1* mutant mice were viable but had reduced fertility. These mice had a Cys mutated to an Ala in exon 6 using the knock-in strategy. All the animals were maintained in a standard 12-h light/dark cycle (lights on at 6:00 a.m.) with ad libitum access to a standard laboratory diet. The animals were euthanized by cervical dislocation, and the corneas were rapidly harvested afterwards.

### Corneal epithelial wound healing model

Central corneal abrasion was performed as described previously^5^. Briefly, animals were anesthetized by i.p. injection of pentobarbital (50 mgkg^−1^ body weight). Under a stereomicroscope, a 2-mm-diameter section of the central corneal epithelium was marked with a trephine and then mechanically scraped using a microspud (Accutome Inc., Malvern, PA, USA). Wound closure was monitored with fluorescein staining of the cornea at 6-h intervals until completion. The measurement and digital analysis of the wound area were performed by comparing the pixels in the histogram function in Adobe Photoshop CS6 (Adobe Systems Inc., San Jose, CA, USA).

### ATSE model

The tobacco smoke exposure protocol was adapted and modified from a sidestream tobacco smoke model previously described by Kojima et al^19^. Briefly, the mice were placed in a homemade smoking chamber (60 × 40 × 35 cm^3^) with continuous fresh-air ventilation (15 mL s^−1^). Lit tobacco cigarettes (about three over a period of approximately 90 min; Marlboro, Philip Morris Tobacco, USA), each containing 11 mg of tar and 1 mg nicotine, were placed into the smoking chamber before corneal abrasion. Subsequently, the same 90-min smoke exposure protocol was repeated once more every 12 h after injury until the end of the experiment, except for two sessions of stimulation (before abrasion and 12 h after abrasion) for measuring epinephrine and norepinephrine in peripheral blood. All the smoke exposure experiments were started at 6:00 p.m. to avoid circadian influence on the wound healing process^68^. The control animals were exposed to RA only.

### Chemical sympathectomy

Chemical sympathectomy (adrenergic denervation) was performed by three i.p. injections of 100 mg kg^−1^ 6-OHDA (Sigma-Aldrich, St. Louis, MO, USA) freshly dissolved in phosphate-buffered saline (PBS). Each injection was administered 2 days apart, and corneal abrasion and/or ATSE were performed 3 days after the final injection. If corneal abrasion is considered day 0, 6-OHDA injections were performed on days 3, 5, and 7. This treatment regimen has previously been found to efficiently sympathectomize animals^52^. The control animals received PBS (vehicle) only. The effect of 6-OHDA on corneal wound healing without ATSE was documented in Supplementary Figure 8 as the control data.

### Agonist/antagonist treatments

Immediately post wounding, the mice were topically administered the selective β2-AR agonist isoepinephrine (5 µL, 0.05 molL^−1^; Tocris Bioscience, UK), the selective β2-AR antagonist timolol (5 µL, 0.01 molL^−1^; Tocris Bioscience, UK), the α1-AR antagonist tamsulosin (5 µL, 20 molL^−1^; Tocris Bioscience, UK). The aforementioned drugs were dissolved in a balanced salt solution (BSS), and the BSS was administered as the vehicle for the control mice. In some experiments, the mice received i.p. injections of the chemokine receptor CXCR2 antagonist SB 225002 (Tocris, 1 molL^−1^, using dimethyl sulfoxide as vehicle) to inhibit neutrophil trafficking 5 min before ATSE^39^. The effect of isoepinephrine, timolol, tamsulosin, and SB 225002 on corneal wound healing without ATSE was documented in Supplementary Figure 8 as the control data.

### Immunostaining of whole-mount corneas

Immunofluorescence staining of whole-mount corneas was performed as described previously^3–6,83^. In brief, eyeballs were enucleated from the mice (following euthanasia by cervical dislocation) at different time points after abrasion and fixed in 4% paraformaldehyde for 2 h. The isolated corneas were washed with PBS three times (once every 5 min) and permeabilized with 0.1% Triton X-100. They were then incubated overnight at 4 °C in anti-mouse Ly6G conjugated with FITC (dilution 1:200) to label neutrophils and in anti-mouse γδ T cell receptor conjugated with PE to label γδ T cells. The controls, using IgG isotype- and species-matched antibodies, were negative in all cases. After incubation with PBS three times, radial cuts were made in each cornea so that it could be flattened by a coverslip, and the cornea was mounted in Fluoromount aqueous mounting medium (Catalog #SLBW7464, Sigma-Aldrich, St. Louis, MO, USA) containing 1 μM of 4′,6-diamidino-2-phenylindole (DAPI; Sigma Chemical, St. Louis, MO, USA) to assess nuclear morphology. Image analysis and quantification of the wounded corneas were performed using a DeltaVision Elite Microscopy Imaging System (GE Healthcare Biosciences, PA, USA), as described previously^7,68,83,84^. Details regarding the antibodies are given in Supplementary Table 1.

### Analysis of dividing cells and infiltrated leukocytes

Divided epithelial cells were counted in nine microscopic fields across the cornea from limbus to limbus, as previously described (Supplementary Figure 4);^8,44^ each field (approximately 0.53 mm in diameter) was viewed using a ×40 objective lens. To compare the difference in neutrophils (Ly6G^+^ cells) infiltrating the wound area between groups, the average number of neutrophils in four central fields (Supplementary Figure 4) was recorded as the influx of neutrophils into a representative wounded cornea. The neutrophil number in the whole-corneal thickness was counted manually in a stepwise fashion by adjusting the scroll arrow of the dialog box in the opened file with the DeltaVision imaging system. To compare the difference in γδ T cells (GL3^+^ cells) infiltrating the wounded cornea between different groups, the average number of cells in nine fields from limbus to limbus (×40; Supplementary Figure 4) was recorded as the influx of γδ T cells into a representative wounded cornea.

### RNA extraction and qRT-PCR

As previously described^83^, the corneas were cut into small pieces and then homogenized with TissueRuptor (Qiagen, Germantown, MD, USA) in Buffer RZ (Catalog #RK145, Tiangen, China). Total RNA was isolated using the commercially available RNASimple Total RNA Kit (Catalog #DP419, Tiangen, China) and then reversed into complementary DNA (cDNA) using the ReverTra Ace qPCR RT Kit (Catalog #FSQ-101, Toyobo, Osaka, Japan). Finally, the expression level of the target gene in the cDNA was quantified and analyzed with Thunderbird SYBR qPCR Mix (Catalog #QPS-201, Toyobo, Japan). Primer sequences used for each solution are listed in Supplementary Table 2.

### Measurement of plasma epinephrine and norepinephrine

Blood samples were collected by retro-orbital sinus puncture from smoke-exposed mice at different times and centrifuged. One hundred microliters of plasma was separated from the whole blood and stored at −80 °C. According to the manufacturer’s instructions, enzyme-linked immunosorbent assay (ELISA) Kits for epinephrine/adrenaline (Catalog #CSB-E08679m, CUSABIO and CusAb, China) and norepinephrine (Catalog #CSB-E07870m, CUSABIO and CusAb, China) were used to measure the plasma CCh concentration.

### Neutralizing antibody administration

To analyze the local effect of anti-IL-6 and IL-17A on corneal wound healing and inflammation after corneal abrasion, some mice received anti-mouse IL‑6 monoclonal antibody or anti-mouse IL-17A (5 µL, 200 μgmL^−1^) dissolved in PBS, in the form of eye drops, after corneal abrasion every 6 h until the end of the experiments, as described previously^4^. The control animals received an equal concentration of rat IgG1 isotype control as eye drops. Details regarding the antibodies are given in Supplementary Table 1. The effect of mAb IL-6 and mAb IL-17A on corneal wound healing without ATSE was documented in Supplementary Figure 8 as the control data.

### Flow cytometry

For the flow cytometric analysis of peripheral blood, whole blood was drawn by retro-orbital sinus puncture, treated with RBC lysis buffer, stained with antibodies (APC-CD45, PE-anti-GL3, FITC-Ly6G, and Percp-cy5.5-CD11b), gated for singlet cells, and then plotted on a dot-plot of different groups. Flow cytometric analysis of the injured cornea was performed as described previously^83,84^. At selected time points post wounding, corneas were harvested from C57BL/6J mice treated with RA or ATSE, dissected, and incubated for 1.5–2 h at 37 °C in 0.2% collagenase type I (Catalog #C0130, Sigma-Aldrich, St. Louis, MO, USA). Tissue from 20 corneas was pooled for each batch. The cell pellet was resuspended at room temperature for 10 min with Flow Cytometry Staining Buffer (Catalog #00-4222, eBioscience, Waltham, MA, USA) containing anti-mouse CD16/32 antibody and then stained with one of the following antibodies at room temperature: anti-mouse CD11b antibody conjugated with PE-Cyanine7 or anti-mouse GL3 antibody conjugated with PE. For Ki-67 or IL-17A intracellular staining, cells were incubated for 20 min with mouse anti-Ki-67 conjugated with APC or BV421 Rat Anti-mouse IL-17A. The data were analyzed using FlowJo V10. Isotype controls are given in Supplementary Figure 7. Details regarding the antibodies are given in Supplementary Table 1.

### Sorting and transcript amplification of inflammatory cells

Corneal injury and immune cell sorting from injured corneas were performed as described previously^7,84^. In brief, the mechanically injured corneas from 12 C57BL/6J mice (*n* = 6) were removed 18 h after injury. Single-corneal-cell suspensions were harvested after collagenase digestion. The cells were counted and resuspended in FACS buffer (PBS + 0.5% bovine serum albumin + 0.01% NaN_3_) at 4 °C at a concentration of 1 × 10^7^ cells mL^−1^ in a sterile FACS tube (5-mL polystyrene round-bottom tube). We proceeded with the Fc-blockade by adding anti-CD16/CD32 (clone 93, Catalog #14-0161-81, eBioscience) and incubating for 15 min at 4 °C in the dark. For neutrophil sorting, APC-conjugated anti-CD45 (clone 30-F11, Catalog #559864, BD Biosciences), PE-conjugated anti-Ly6G (clone 1A8, Catalog #551481, BD Biosciences), and APC-Cy7-conjugated anti-CD11b (clone M1/70, Catalog #47-0112-82, eBioscience) were added and incubated for 30 min at 4 °C in the dark. Excess antibodies were washed off twice by adding 1 mL FACS buffer to each tube and centrifuging for 7 min at 472 × *g* at 4 °C. The cell pellet was resuspended at a concentration of 5 × 10^6^ cells mL^−1^ in sorting buffer (calcium- and magnesium-free PBS supplemented with 10% FBS and 0.5 mM EDTA). CD45^+^ Ly6G^+^ CD11b^+^ cells were sorted using previously described sorting procedures^85^. For γδ T cell sorting, APC-conjugated anti-CD45 (clone 30-F11, Catalog #559864, BD Biosciences) and PE-conjugated γδ T cell receptor (Clone GL3, Catalog #561997, BD Biosciences) were added. Other procedures were performed according to the neutrophil sorting protocol as described above. Finally, the REPLI-gWTA Single Cell Kit (Qiagen; no. 150063) was used for transcript amplification of small numbers of neutrophils and γδ T cells, as described before^7,29^.

### Statistical analysis

All the data are expressed as the mean ± standard deviation (SD). Based on the data set, unpaired Student’s *t* test, one-way analysis of variance (ANOVA) followed by Fisher’s least significant difference post hoc test, two-way or repeated-measures two-way ANOVA followed by Bonferroni or Sidak’s post hoc were performed. GraphPad Prism (GraphPad Software, La Jolla, CA, USA) and SPSS 21.0 (IBM, USA) software were used to perform all the statistical analyses. A *p* value of <0.05 was considered significant.

## Supplementary information

Description of Supplementary Data

Supplementary information

Supplementary data 1

Supplementary data 2

Supplementary data 3

Supplementary data 4

Supplementary data 5

Supplementary data 6

Supplementary data 7

Supplementary data 8

Supplementary data 9

## Data Availability

The data source underling the graphs in the main figures is available in Supplementary Data 1–9. All the data supporting the findings of this study are available from the authors upon request.
