# Supplementary material for: Acute tobacco smoke exposure exacerbates the inflammatory response to corneal wounds in mice via the sympathetic nervous system
Source: Commun Biol. 2019 Jan 24;2:33. doi: 10.1038/s42003-018-0270-9 (PMC6345828; doi:10.1038/s42003-018-0270-9)
Supplement: Supplementary file 1 — Description of Supplementary Data [file 42003_2018_270_MOESM1_ESM.docx]

**Description of Additional Supplementary Files**

**File Name**: Supplementary Data 1

**Description**: **Figure 1. Acute tobacco smoke exposure (ATSE) impairs corneal wound healing and exacerbates inflammation.**

Figure 1B Percent decrease in open wound area over time post-wounding

Figure 1C Change over time in number of dividing epithelial cells after wounding

Figure 1D Neutrophil influx into cornea over time after corneal abrasion

Figure 1E γδ T cell influx into wounded cornea over time after corneal abrasion

Figure 1F Representative flow cytometry plots of CD11b+ Ly6G+ neutrophils

Figure 1G Representative flow cytometry plots of GL3+ γδ T cells

Figure 1I Relative expression of NF-kB measured by qRT-PCR in whole corneal mRNA at 6, 12, 18, and 24 h after abrasion

Figure 1J Relative expression of IL-6 measured by qRT-PCR in whole corneal mRNA at 6, 12, 18, and 24 h after abrasion

Figure 1K Relative expression of IL-17A measured by qRT-PCR in whole corneal mRNA at 6, 12, 18, and 24 h after abrasion

**File Name**: Supplementary Data 2

**Description**: **Figure 2. ATSE enhances SNS signaling activity and mobilization of neutrophils and γδ T cells into the circulation and corneal limbus.**

Figure 2A ELISA-measured plasma epinephrine concentration after corneal abrasion in RA, ATSE, and ATSE + 6-OHDA treatments

Figure 2B ELISA-measured plasma norepinephrine concentration after corneal abrasion in RA, ATSE, and ATSE + 6-OHDA treatments

Figure 2D Cumulative neutrophil (CD11b+Ly6G+) percentage among total leukocytes in peripheral blood 1 h after ATSE

Figure 2F Cumulative percentage of γδ T cells (GL3+) among total leukocytes in peripheral blood 1 h after ATSE

Figure 2H Quantification of neutrophil recruitment to corneal limbus 1 h after RA, ATSE-only, and 6-OHDA + ATSE treatments

Figure 2J Quantification of γδ T cell recruitment to corneal limbus 1 h after RA, ATSE-only, and 6-OHDA + ATSE treatments

**File Name**: Supplementary Data 3

**Description**: **Topical administration of a β-adrenergic agonist in the RA group worsens corneal wound healing and inflammation.**

Figure 3B Percent decrease in open wound area over time post-wounding

Figure 3C Number of dividing epithelial cells over time after wounding

Figure 3D Neutrophil influx into the wounded area over time after corneal abrasion

Figure 3E γδ T cell influx into the wounded cornea over time after corneal abrasion

Figure 3F Relative expression of NF-kB measured using mRNA isolated from whole wounded corneas of the RA, ATSE, and RA + isoepinephrine groups at 6, 12, 18, and 24 h after corneal abrasion

Figure 3G Relative expression of IL-6 measured using mRNA isolated from whole wounded corneas of the RA, ATSE, and RA + isoepinephrine groups at 6, 12, 18, and 24 h after corneal abrasion

Figure 3H Relative expression of IL-17A measured using mRNA isolated from whole wounded corneas of the RA, ATSE, and RA + isoepinephrine groups at 6, 12, 18, and 24 h after corneal abrasion

**File Name**: Supplementary Data 4

**Description**: **SNS denervation alleviates ATSE-induced corneal wound healing impairment, as does topical β-adrenergic receptor (AR) antagonist administration.**

Figure 4B Percent decrease in open wound area over time after corneal abrasion in RA or ATSE mice pretreated with 6-OHDA or vehicle

Figure 4C Number of dividing epithelial cells over time post-abrasion in RA or ATSE mice with 6-OHDA or vehicle pretreatment

Figure 4D Neutrophil influx into cornea over time post-abrasion in RA or ATSE mice after 6-OHDA or vehicle pretreatment

Figure 4E γδ T cell influx into limbus post-abrasion in RA or ATSE mice pretreated with 6-OHDA or vehicle

Figure 4F Relative expression of adrenergic receptor mRNAs in flow-gating-sorted neutrophils as CD45+CD11b+Ly6G+ from 20 pooled whole wounded corneas 18 h after injury

Figure 4H Percent decrease in open wound area over time after corneal abrasion in RA or ATSE mice treated with timolol or vehicle

Figure 4I Number of dividing epithelial cells over time post-abrasion in RA or ATSE mice with timolol or vehicle treated

Figure 4J Neutrophil influx into cornea over time post-abrasion in RA or ATSE mice with timolol or vehicle treated

Figure 4K γδ T cell influx into cornea over time post-abrasion in RA or ATSE mice with timolol or vehicle treated

Figure 4L Relative expression of NF-kB measured using mRNA isolated from whole wounded corneas of the RA, ATSE, and RA + Timolol groups at 6, 12, 18, and 24 h after corneal abrasion

Figure 4M Relative expression of IL-6 measured using mRNA isolated from whole wounded corneas of the RA, ATSE, and RA + Timolol groups at 6, 12, 18, and 24 h after corneal abrasion

Figure 4N Relative expression of IL-17A measured using mRNA isolated from whole wounded corneas of the RA, ATSE, and RA + Timolol groups at 6, 12, 18, and 24 h after corneal abrasion

**File Name**: Supplementary Data 5

**Description**: **Figure 5. ATSE-induced impairment of corneal wound healing requires NF-κB signaling.**

Figure 5A Relative NF-κB expression using mRNA isolated from whole wounded corneas of the RA, ATSE, and ATSE + 6-OHDA groups at 6, 12, 18, and 24 h after corneal abrasion

Figure 5C Percent decrease in open wound area over time after wounding

Figure 5D Dividing epithelial cells over time post-wounding

Figure 5E Neutrophil influx into the cornea over time after corneal abrasion

Figure 5F γδ T cell influx into wounded corneas over time

**File Name**: Supplementary Data 6

**Description**: **Figure 6.** **Effects of IL-6 neutralization (using IL-6 antibody mAb IL-6) on ATSE-induced wound healing delay and exacerbated inflammation.**

Figure 6A Relative expression of IL-6 using mRNA isolated from whole wounded corneas of RA, ATSE, and ATSE + 6-OHDA mice at 6, 12, 18, and 24 h after corneal abrasion

Figure 6C Percent decrease in open wound area over time after corneal wounding of RA or ATSE mice treated with mAb IL-6 or isotype IgG as a control

Figure 6D Number of dividing epithelial cells over time after wounding in RA or ATSE mice treated with mAb IL-6 or isotype IgG control

Figure 6E Neutrophil influx into the cornea over time after corneal abrasion in RA or ATSE mice treated with mAb IL-6 or isotype IgG control

Figure 6F γδ T cell influx into injured cornea over time after corneal abrasion in RA or ATSE mice treated with mAb IL-6 or isotype IgG control

**File Name**: Supplementary Data 7

**Description**: **Figure 7. IL-17A is a critical mediator of ATSE-induced delayed wound healing and exacerbated inflammation.**

Figure 7A Relative IL-17A expression using mRNA isolated from whole wounded corneas of the RA, ATSE, and ATSE + 6-OHDA groups at 6, 12, 18, and 24 h post-corneal abrasion

Figure 7C Percent decrease in open wound area over time post-wounding

Figure 7D Number of dividing epithelial cells over time post-wounding

Figure 7E Neutrophil influx into the cornea over time after corneal abrasion

Figure 7F γδ T cell influx into the cornea over time after corneal abrasion

**File Name**: Supplementary Data 8

**Description**: **Figure 8. The α1a-AR antagonist tamsulosin alleviates delayed wound healing and exacerbated inflammation.**

Figure 8A Relative expression of adrenergic receptors in sorted CD45+GL3+ γδ T cells from 20 pooled wounded corneas 18 h after corneal abrasion

Figure 8C Percent decrease in open wound area over time after wounding in the RA, ATSE, or ATSE + tamsulosin treated mice

Figure 8D Epithelial cell division over time after wounding in the RA, ATSE and ATSE + tamsulosin groups

Figure 8E Neutrophil influx into the cornea over time after injury in RA, ATSE and ATSE + tamsulosin groups

Figure 8F γδ T cell influx into the wounded cornea over time after corneal abrasion in the RA, ATSE and ATSE + tamsulosin groups

Figure 8G Whole-cornea mRNA expression of *NF-kB* in injured corneas of the RA, ATSE, and ATSE + topical tamsulosin treated groups at 6, 12, 18, and 24 h after abrasion

Figure 8H Whole-cornea mRNA expression of *IL-6* in injured corneas of the RA, ATSE, and ATSE + topical tamsulosin treated groups at 6, 12, 18, and 24 h after abrasion

Figure 8I Whole-cornea mRNA expression of *IL-17A* in injured corneas of the RA, ATSE, and ATSE + topical tamsulosin treated groups at 6, 12, 18, and 24 h after abrasion

**File Name**: Supplementary Data 9

**Description**: **Figure 9. Interaction and feedback among IL-6, IL-17A, and NF-κB after ATSE**

Figure 9A Kinetics of the relative expression of NF-κB, IL-17A, and IL-6 using mRNA isolated from whole wounded corneas in the RA group at 6, 12, 18, and 24 h after corneal abrasion.

Figure 9B Effects of genetic NF-κB deactivation on the expression of IL-17A at different time points after corneal abrasion in the RA and ATSE groups.

Figure 9C Effects of local IL-6 neutralization (with IL-6 antibody mAb IL-6) on the expression of IL-17A at different time points after corneal abrasion in the RA and ATSE groups.

Figure 9D Effects of NF-κB genetic deactivation on the local expression of IL-6 at different timepoints after corneal abrasion in the RA and ATSE groups.

Figure 9E Effects of local IL-6 neutralization (with mAb IL-6) on the local expression of NF-κB at different time points after corneal abrasion in the RA and ATSE groups.

Figure 9F Effects of local IL-17A neutralization on the mRNA expression of whole corneal NF-κB1 at different time points after corneal abrasion in the RA and ATSE groups after wounding.

Figure 9G Effects of local IL-17A neutralization (using IL-17A antibody mAb IL-17A) on the gene expression of IL-6 in the whole cornea at different time points after corneal abrasion in the RA and ATSE groups. n=4 corneas per time point in each group.
