# Supplementary material for: Acute tobacco smoke exposure exacerbates the inflammatory response to corneal wounds in mice via the sympathetic nervous system
Source: Commun Biol. 2019 Jan 24;2:33. doi: 10.1038/s42003-018-0270-9 (PMC6345828; doi:10.1038/s42003-018-0270-9)
Supplement: Supplementary file 2 — Supplementary information [file 42003_2018_270_MOESM2_ESM.pdf]

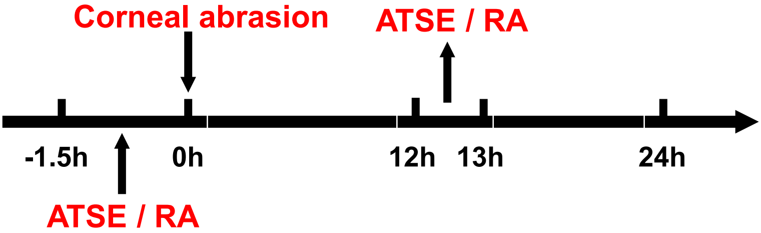

Supplementary Figure 1. Schematic of ATSE treatment strategies in different groups of mice. Animals were treated with ATSE or RA 1.5 hours before corneal abrasion and treated again 12 h after abrasion.

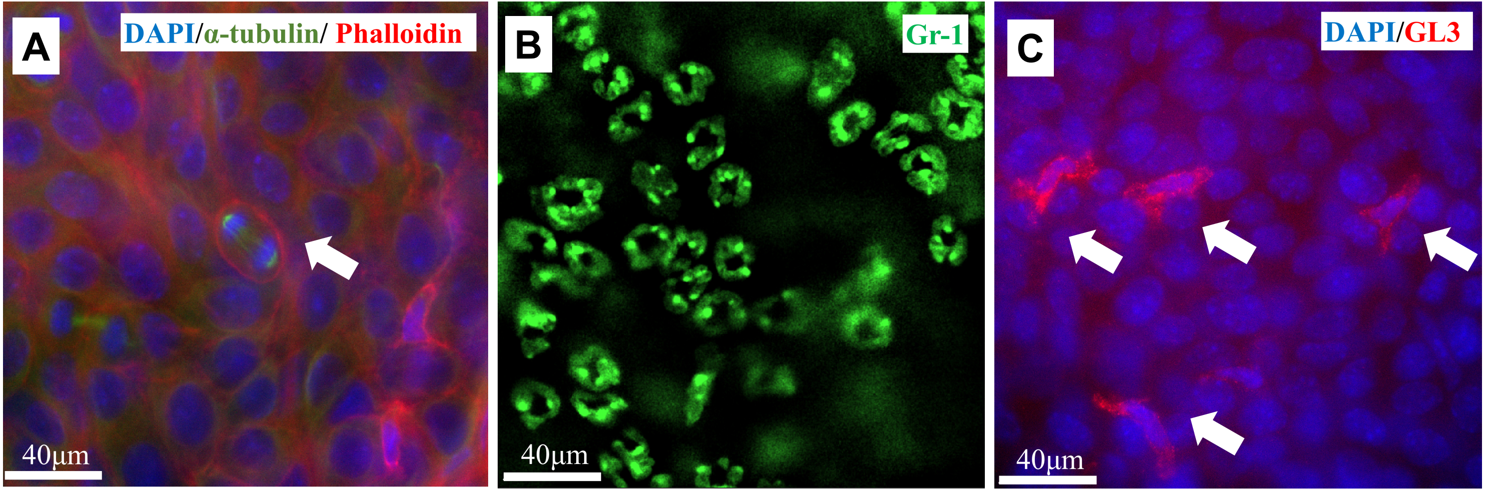

Supplementary Figure 2. Representative images. (A) Representative image of dividing cells (indicated by the white arrow) with staining for DAPI (blue), anti-tubulin α (green) and phalloidin (red). **(B)** Representative image of neutrophils (green) in the wound area (labeled with FITC-Gr-1). **(C)** Representative image of γδ T cells (white arrows) labeled with PE-anti-GL3 in the limbal epithelium.

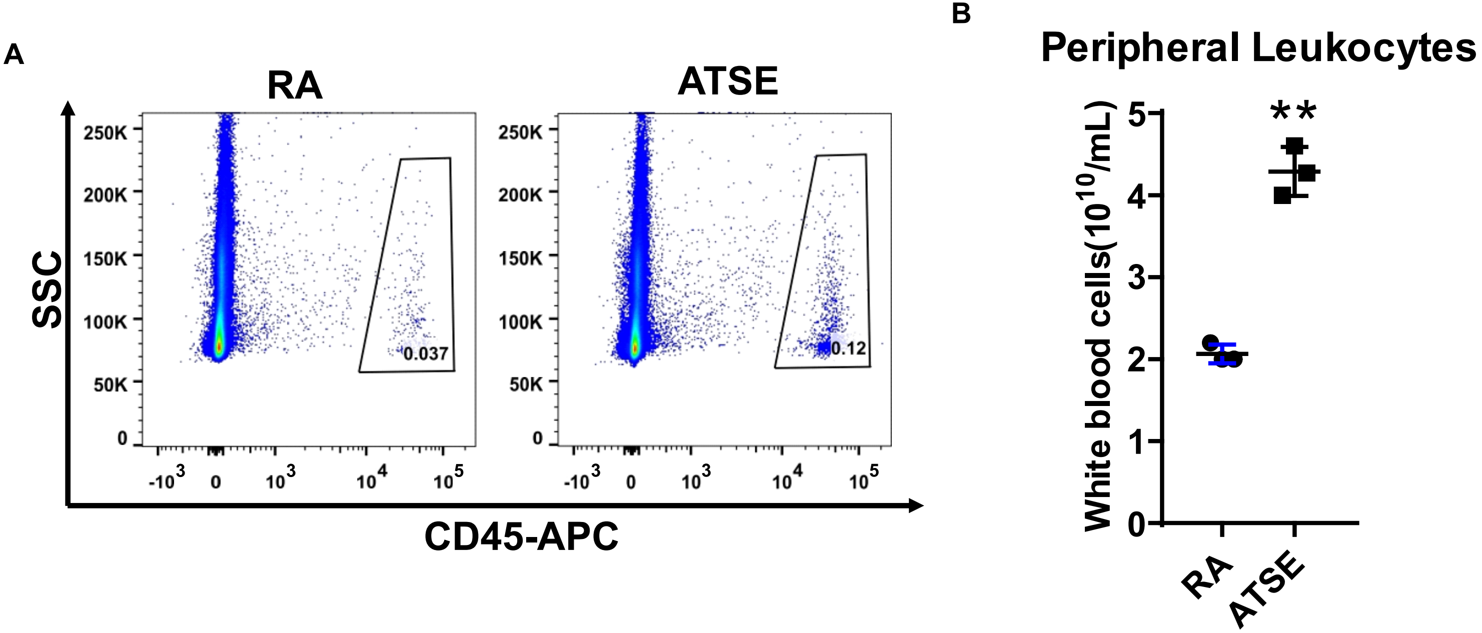

Supplementary Figure 3. Isolation of CD45^{+} cells from peripheral whole blood of unwounded mice. Flow cytometric plots of CD45^{+} cells in the RA and ATSE groups (left-hand plots) and fluorescence-activated cell sorting (FACS) quantification of CD45^{+} cells from peripheral whole blood 1 h after RA or ATSE treatment (histogram on right). ***SSC***, side scatter

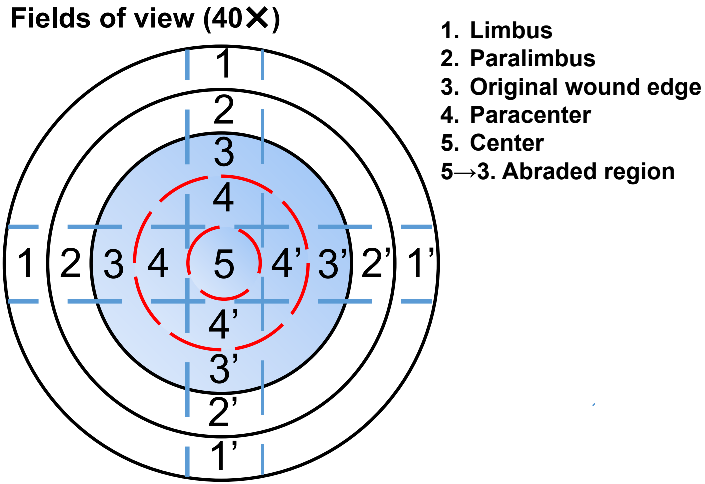

Supplementary Figure 4. Diagram showing the microscopic fields (at 40X) examined during the analysis of the cornea. The figure shows the view under a Deltavision Elite microscope at 40X, as indicated in each of the four regions of the cornea. The analysis was performed by counting specific parameters for each cell type.Ly6G^{+} neutrophils were counted in four Field 4s as averages of the infiltrated neutrophils at different time points after corneal abrasion. γδ T cells (GL3^{+} cells) were counted in the whole thickness of the cornea and mitotic cells from limbus to limbus (total of nine fields: 1, 2, 3, 4, 5, 4’, 3’, 2’, 1’) at different time points after corneal abrasion. The blue area represents the original central wound area with epithelial removal.

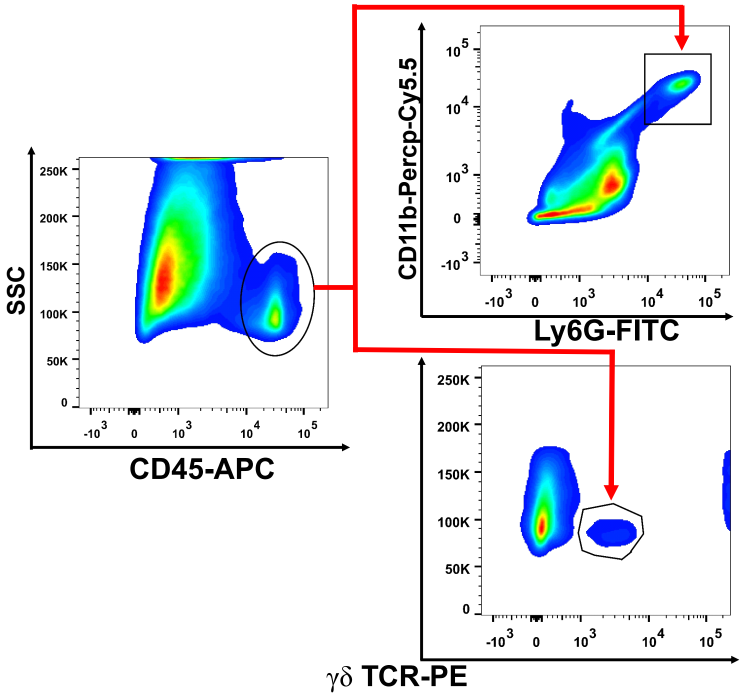

Supplementary Figure 5. Sorting of neutrophils (top) and γδ T cells (bottom) from CD45$^+$ leukocytes (left) of wounded corneas 18 h after abrasion. Flow cytometric analysis of CD45$^+$ cells in the wounded cornea sorted into Ly6G$^+$ CD11b$^+$ neutrophils and γδ T cells.

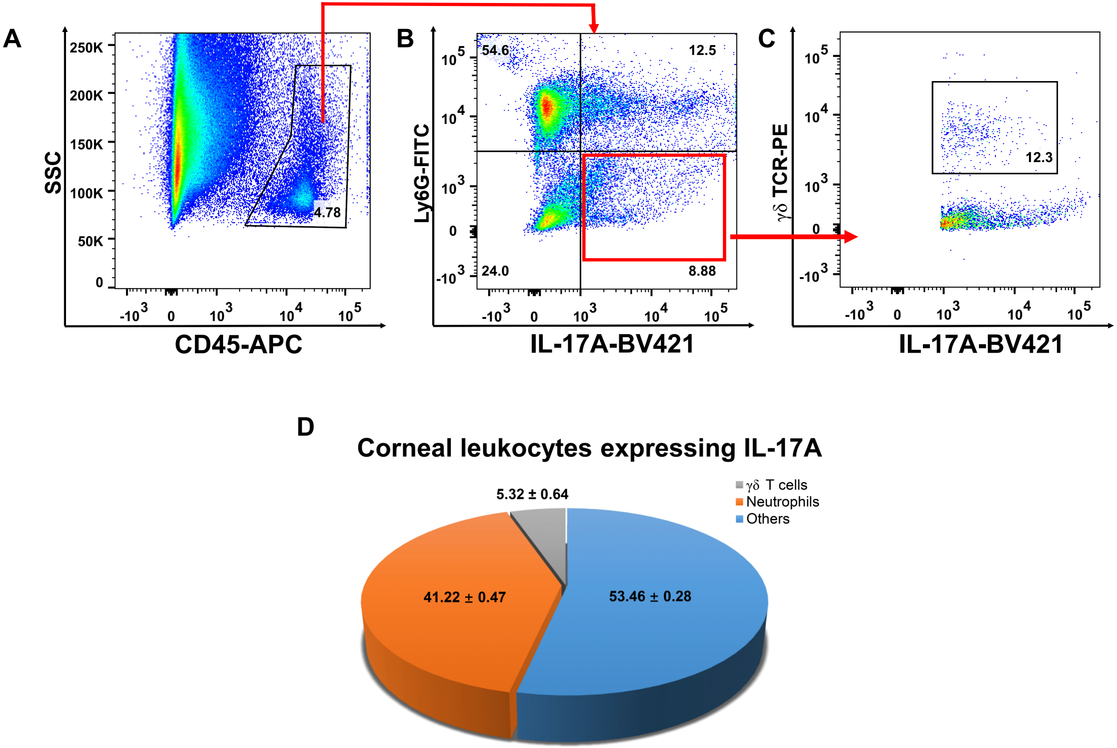

Supplementary Figure 6. Local IL-17A-producing cells recruited to the wounded corneas. (A) Representative staining plots showing gated CD45$^+$ cells (4.78% in black-outlined box in right plot) from a single cell suspension of collagenase-digested wounded corneas 18 h after abrasion. **(B)** Ly6G$^+$ IL-17A$^+$ (12.5% in the upper-right box) and Ly6G$^-$IL-17A$^+$ (8.88% in the lower-right box) cell populations from among the CD45$^+$ cell group. **(C)** GL3$^+$IL-17A$^+$ (12.3 % in the upper box) and GL3$^-$IL-17A$^+$ (87.7% in the lower-right area) cell populations from among the Ly6G$^-$IL-17A$^+$ cell group. **(D)** Pie chart showing the contribution of IL-17A from each cell subset ± SEM.

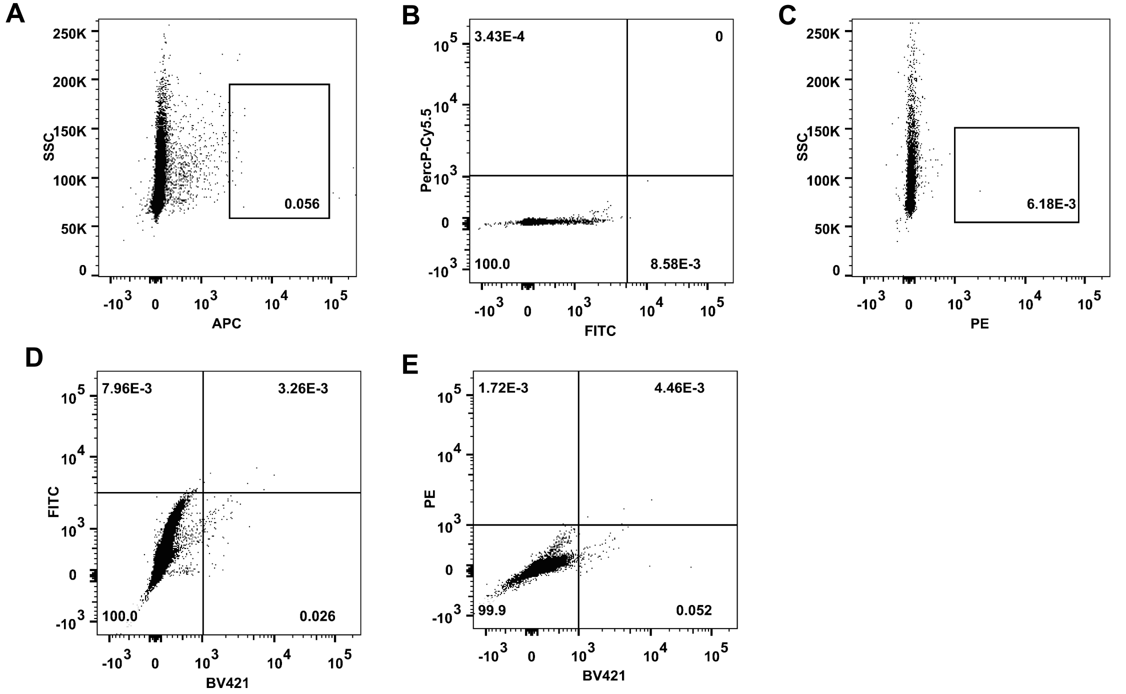

Supplementary Figure 7. Isotype control. **(A)** APC isotype control (CD45). **(B)** FITC-PrecP-cy5.5 isotype control (FITC-Ly6G, PrecP-cy5.5-CD11b). **(C)** PE isotype control (γδ TCR). **(D)** BV421-IL-17A, FITC-Ly6G. **(E)** BV421-IL-17A, PE-γδ TCR.

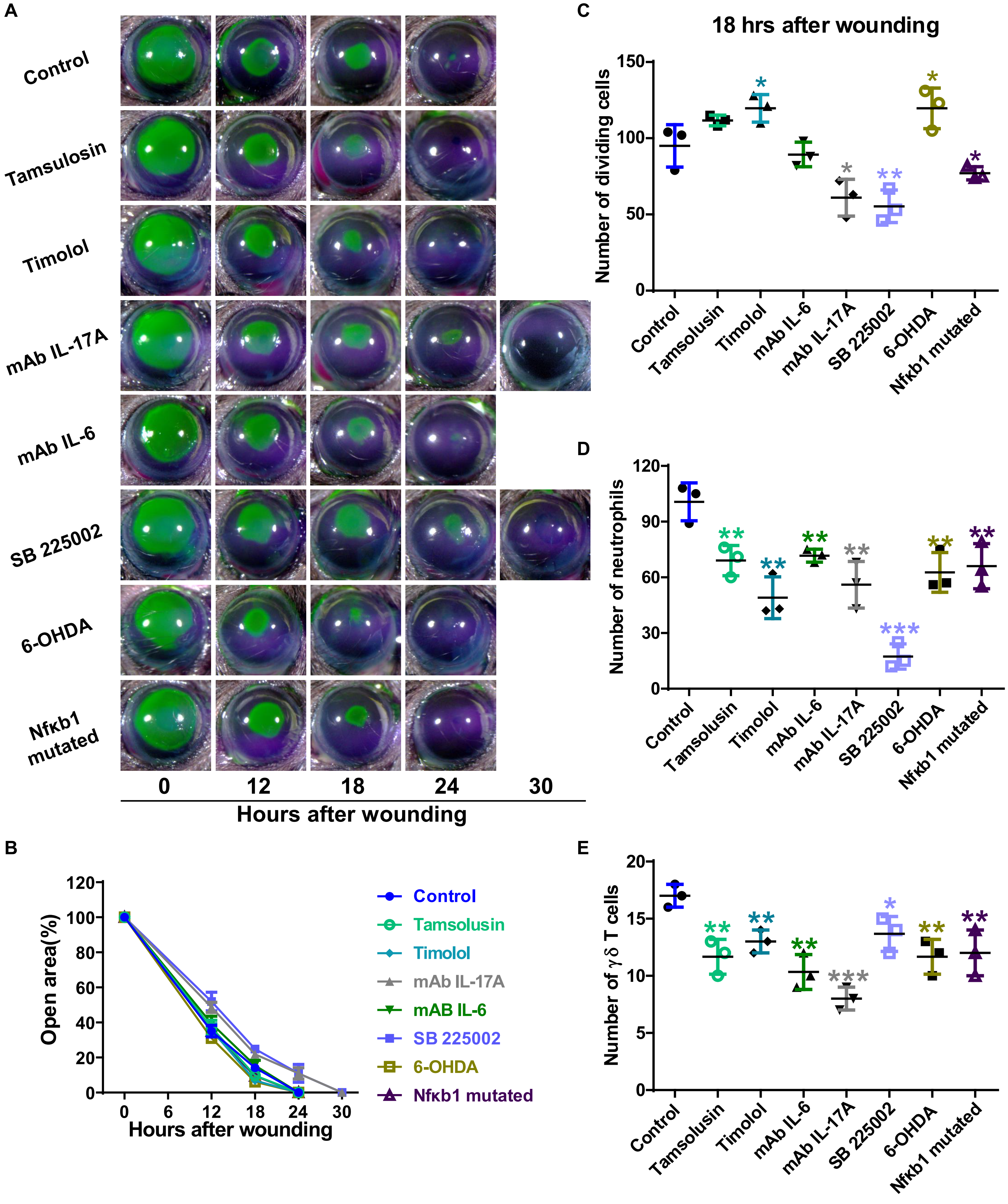

Supplementary Figure 8. The effect of all drugs and antibodies on corneal wound healing without ATSE. **(A)** Representative images of open corneal wounds (revealed by topical fluorescein) and visible wound closure over time post-wounding. **(B)** Percent decrease in open wound area over time after wounding. **(C)** Number of dividing epithelial cells over time post-abrasion. **(D)** Neutrophil influx into the cornea over time post-abrasion. **(E)** γδ T cell influx into the limbus post-abrasion. Student't test, * = $p < 0.05$, ** = $p < 0.01$, and *** $p < 0.001$.

Antibody Name	Company	Catalog NO.	Clone
PE Rat Anti-Mouse CD31	BD Biosciences	553373	MEC 13.3
Anti-α-Tubulin−FITC antibody	Sigma	F2168	DM1A
Rhodamine-phalloidin	Thermo-Fisher Scientific	R415	
APC Anti-mouse Ki-67 Antibody	BioLegend	652405	16A8
BV421 Rat Anti-mouse IL17A	BD Biosciences	566286	TC11-18H10
FITC Rat Anti-Mouse Ly-6G	BD Biosciences	551460	1A8
APC Rat Anti-Mouse CD45	BD Biosciences	559864	30-F11
CD11b Monoclonal Antibody, PerCP-Cyanine5.5	eBioscience	45-0112-82	M1/70
PE Hamster Anti-Mouse γδ T-Cell Receptor	BD Biosciences	553178	GL3
APC Rat IgG2a, κ Isotype Ctrl Antibody	BioLegend	400511	RTK2758
FITC Rat IgG2a, κ Isotype Control	BD Biosciences	553929	R35-95
BV421 Rat IgG1, κ Isotype Control	BD Biosciences	562868	R3-34
FITC Rat IgG2b, κ Isotype Control	BD Biosciences	553988	A95-1
PE Mouse IgG1, κ Isotype Control Antibody	BioLegend	400111	MOPC-21
Rat IgG2b κ Isotype Control, PerCP-Cyanine5.5	eBioscience	45-4031-80	eB149/10H5
APC Rat IgG2b, κ Isotype Control	BD Biosciences	555745	A95-1
Anti-Mouse CD16/CD32	eBioscience	14-0161-86	93
Anti-Mouse IL-17A Functional Grade, Purified	eBioscience	16-7173-85	eBioMM17F3
Mouse IgG1 κ Isotype Control, Functional Grade	eBioscience	16-4714-82	P3.6.2.8.1
Mouse IL-6 Antibody	R&D Systems	MAB406	MP5-20F3
Rat IgG1 Isotype Control	R&D Systems	MAB005	43414

Supplementary Table 1. Antibodies used in this study

ID	Primer Sequence		Temp (°C)
IL-6	Forward	5'-GACTTCCATCCAGTTGCCTT-3'	59
	Reverse	5'-TGTGTAATTAAGCCTCCGACT-3'	
NfκB1	Forward	5'-GGAAGACAAGGAGCAGGACAT-3'	59
	Reverse	5'-AGCGTGGAGGTGGATGATG-3'	
GAPDH	Forward	5'-CAAGGACACTGAGCAAGAG-3'	57
	Reverse	5'-TGCAGCGAACTTTATTGATG-3'	
IL-17A	Forward	5'-ATCTGTGTCTCTGATGCTGTTG-3'	57
	Reverse	5'-GGAACGGTTGAGGTAGTCTGA-3'	
Adrb1	Forward	5'-TTACTCAAGACCGAAAGCAG-3'	55
	Reverse	5'-CCATACTAAGCCACACTCTC-3'	
Adrb2	Forward	5'-CTCCTTTTTGCCTATCCAGA-3'	55
	Reverse	5'-GCACGTAGAAAGACACAATC-3'	
Adra1a	Forward	5'-GTGGAGTTATTGGATCGCTG-3'	55
	Reverse	5'-CTCCAAAGACCAAGACCCAG-3'	
Adra1d	Forward	5'-TATCTGTGGGACCGCTACTA-3'	55
	Reverse	5'-GATCCTCAGAACCACCTCAG-3'	
Adra2b	Forward	5'-GTTCCCCTTCTTCTTCAGCT-3'	55
	Reverse	5'-TTGCCTGCTCAATGACAAAG-3'	
Adra2c	Forward	5'-GTTCCCCTTCTTCTTCAGCT-3'	55
	Reverse	5'-CCTTCTCCTCCTTCGAAAG-3'	

Supplementary Table 2. Primer Sequences
